# Supplementary material for: Interaction-selective molecular sieving adsorbent for direct separation of ethylene from senary C2-C4 olefin/paraffin mixture
Source: Nat Commun. 2024 Jan 20;15:625. doi: 10.1038/s41467-024-45004-9 (PMC10799885; doi:10.1038/s41467-024-45004-9)
Supplement: Supplementary file 1 — Supplementary Information [file 41467_2024_45004_MOESM1_ESM.pdf]

Supplementary Information

Interaction-Selective Molecular Sieving Adsorbent for Direct Separation of Ethylene from Senary C_2-C_4 Olefin/Paraffin Mixture

Yong Peng[1], Hanting Xiong[1], Peixin Zhang[1], Zhiwei Zhao[1], Xing Liu[1], Shihui Tang[1], Yuan Liu[1], Zhenliang Zhu[1], Weizhen Zhou[1], Zhenning Deng[1], Junhui Liu[1], Yao Zhong[1], Zeliang Wu[1], Jingwen Chen[1], Zhenyu Zhou[1], Shixia Chen[1], Shuguang Deng[2], Jun Wang[1*]

1. Chemistry and Chemical Engineering School, Nanchang University, Nanchang, Jiangxi 330031, China

2. School for Engineering of Matter, Transport and Energy, Arizona State University, Tempe, Arizona 85287, United States

*Corresponding authors.

E-mail addresses: jwang7@ncu.edu.cn (J. Wang).

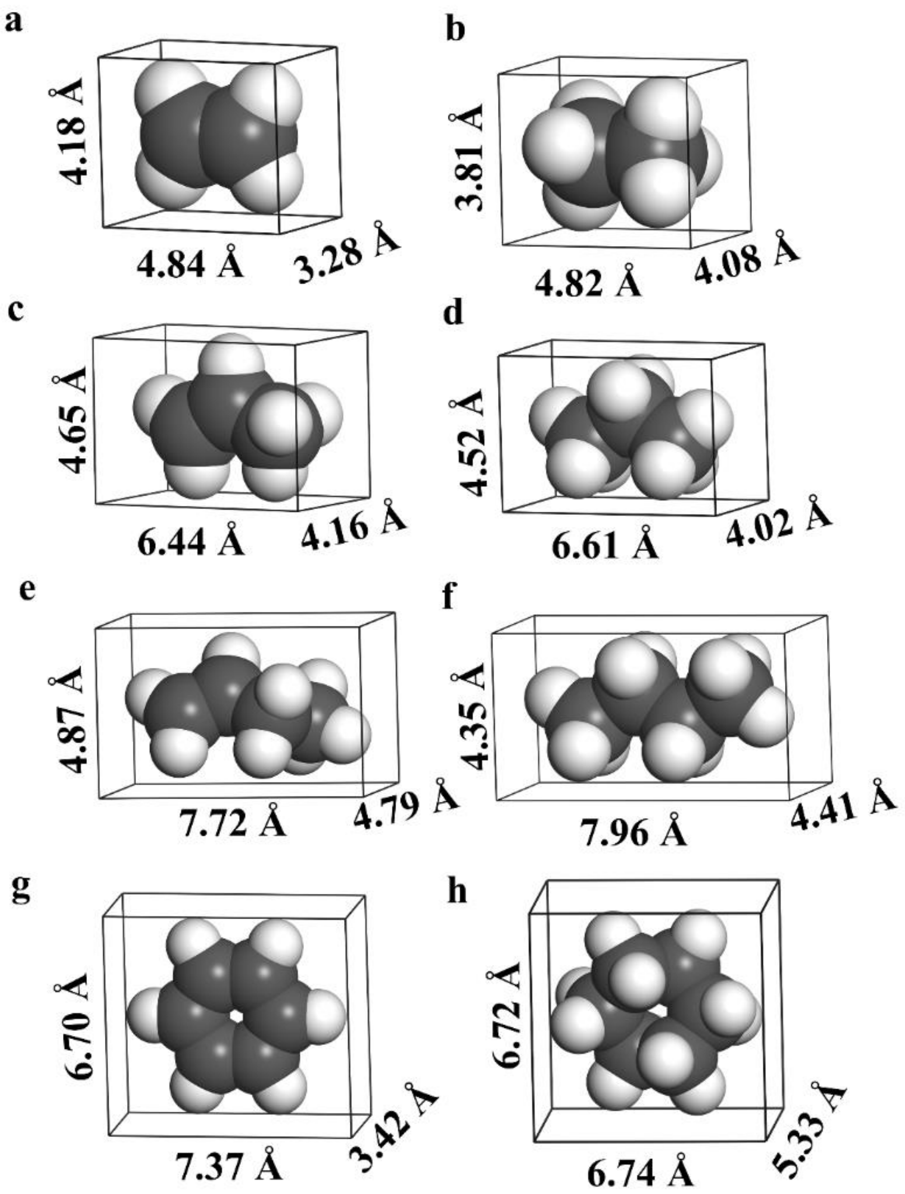

Supplementary Figure 1. Gas molecule structures. Molecular dimensions of (a) C_2H_4 and (b) C_2H_6 (kinetic diameter difference: 0.028 nm, boiling point (b.p) difference: 15 K); (c) C_3H_6 and (d) C_3H_8 (kinetic diameter difference: 0.042 nm, b.p difference: 5 K); (e) n-C_4H_8 and(f) n-C_4H_{10} (kinetic diameter difference: 0.053 nm, b.p difference: 5.8 K); (g) benzene and (h) cyclohexane (kinetic diameter difference: 0.07 nm, b.p difference: 0.6 K).

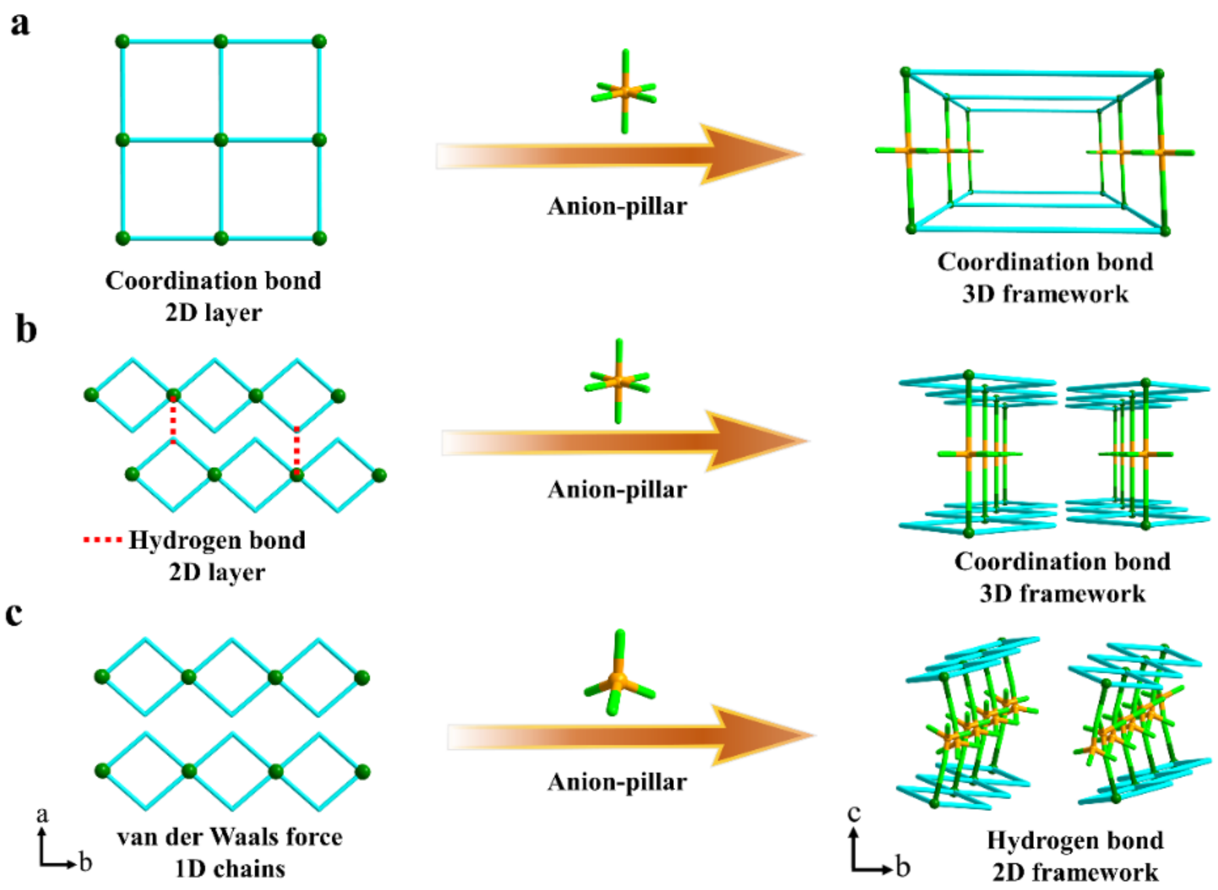

Supplementary Figure 2. Schematic of strategy for preparing hybrid MOF adsorbents. (a) two-dimensional square networks (connected by coordination bond) pillared by anions with rigid framework; (b) one-dimensional networks (connected by H-bond) pillared by anions with rigid-flexible framework; (c) one-dimensional networks (connected by van der Waals force) pillared by anions with more flexibility.

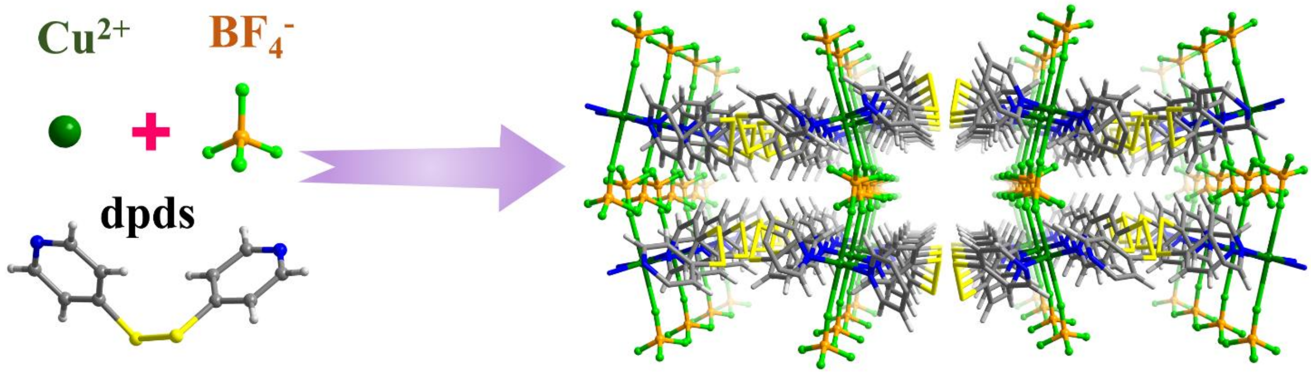

Supplementary Figure 3. Schematic of the synthesis process. Schematic diagram of the synthesis process of BFFOUR-Cu-dpds.

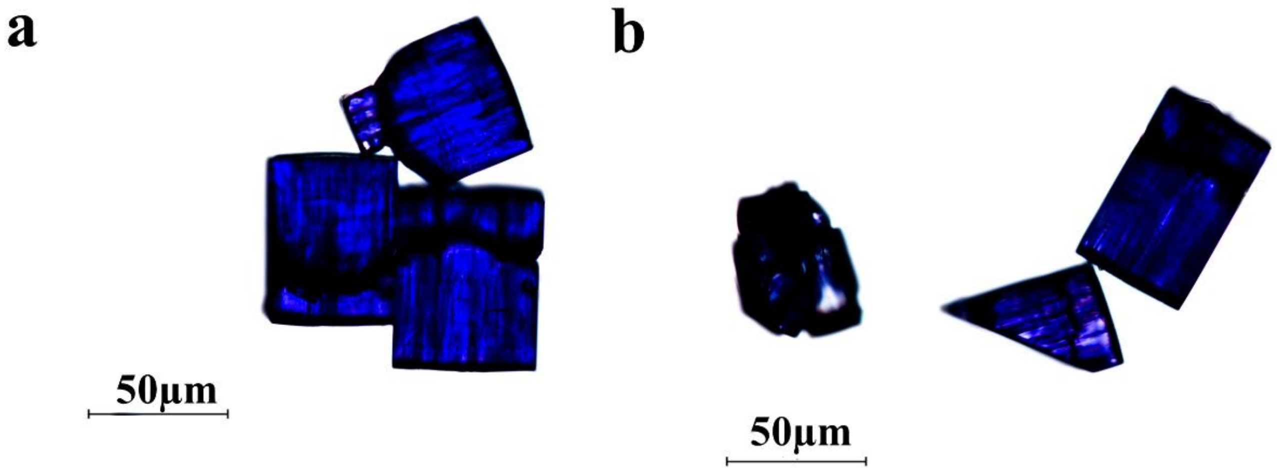

Supplementary Figure 4. The digital photos of single-crystal of BFFOUR-Cu-dpds. The digital image of BFFOUR-Cu-dpds crystals (a) before and (b) after activation.

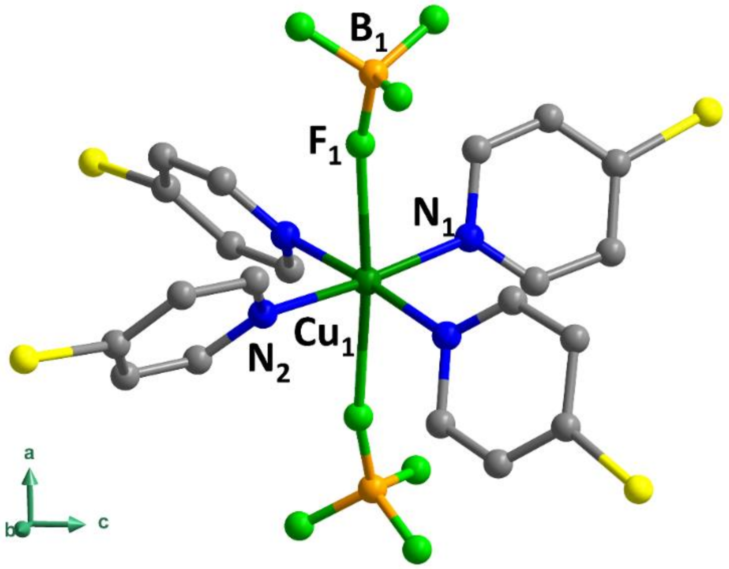

Supplementary Figure 5. The coordination environment of Cu^{2+} in BFFOUR-Cu-dpds. The rest of dpds and H atoms are omitted for clarity. Dark green = Cu, light green = F, grey = C, blue = N, orange = B, yellow = S. Selected bond lengths: Cu_1-N_1 = 2.0284(129) Å, Cu_1-N_2 = 2.0414(116) Å, Cu_1-F_1 = 2.4125(161) Å. Selected bond angles: Cu_1-F_1-B_1 = 166.818(1282)°, N_1-Cu_1-F_1 = 89.205(567)°, N_2-Cu_1-F_1 = 85.728(564)°.

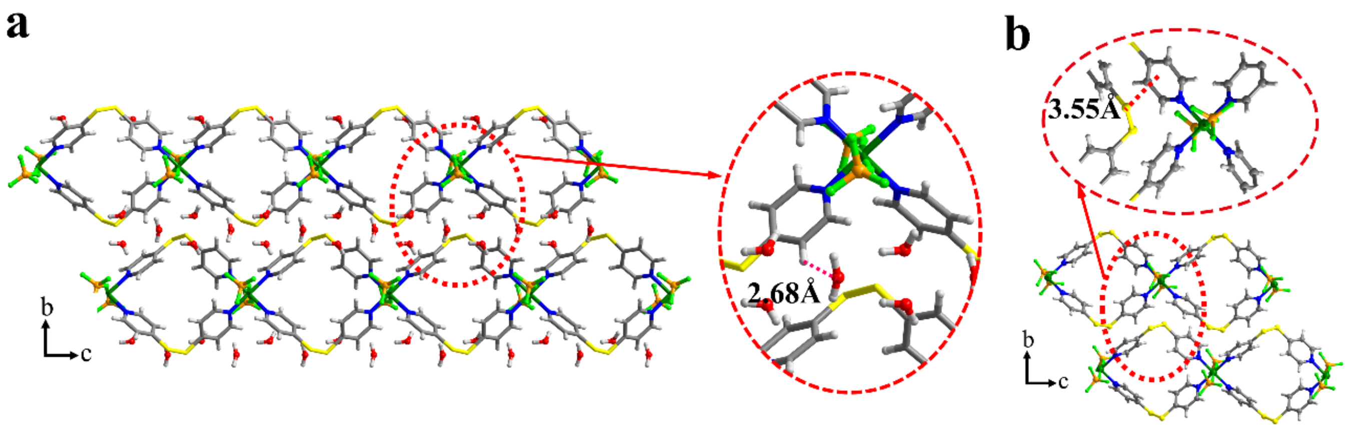

Supplementary Figure 6. The structural diagram of BFFOUR-Cu-dpds containing H_2O molecules. (a) The crystal structure of as-synthesized BFFOUR-Cu-dpds viewed along *a* axis. H_2O molecules are trapped in the interlayer space with O···H hydrogen bonds (2.68 Å); (b) The distances and interactions between layers, S···phenyl plane (3.55 Å).

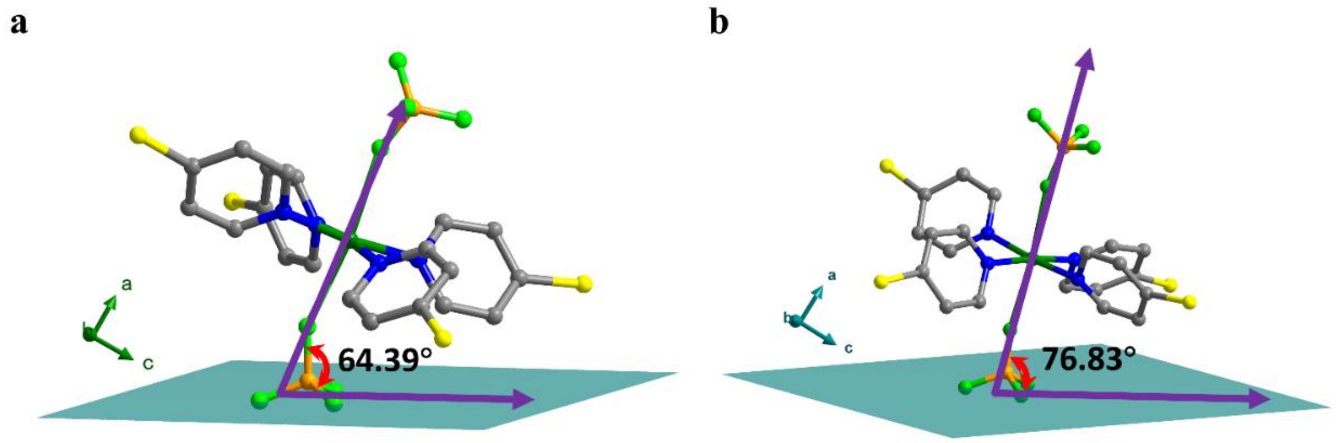

Supplementary Figure 7. BF_4^- deflection angle diagram of BFFOUR-Cu-dpds. The dihedral angle of BF_4^- in (a) as-synthesized and (b) activated BFFOUR-Cu-dpds along *a* axis.

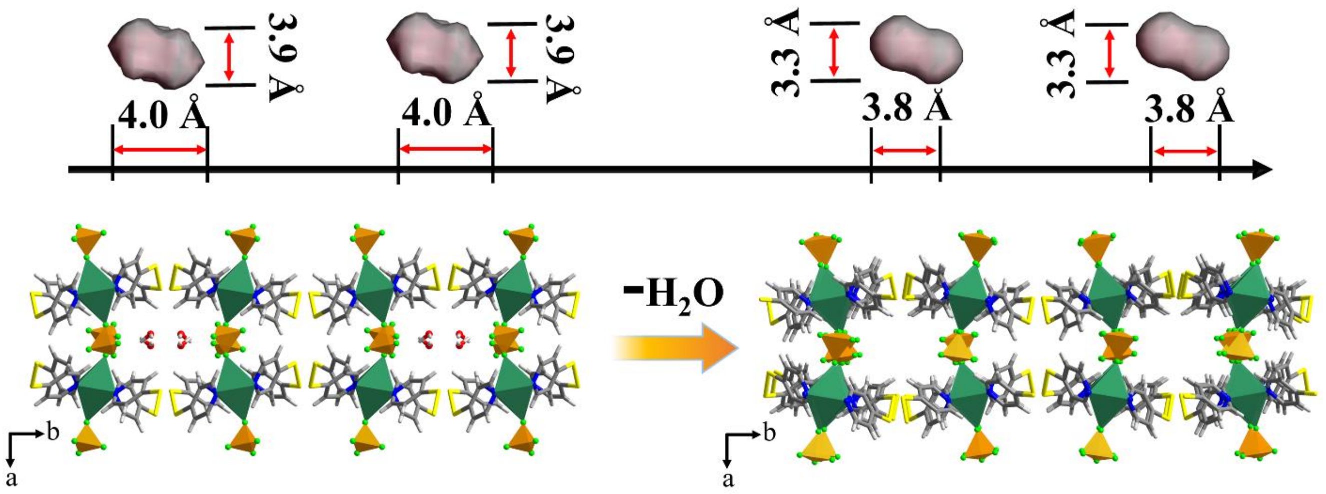

Supplementary Figure 8. The pore sizes before and after activation viewed along *c* axis. Color code: Cu, dark green; B, orange; F, light green; C, dark grey; H, light grey; S, yellow; N, blue.

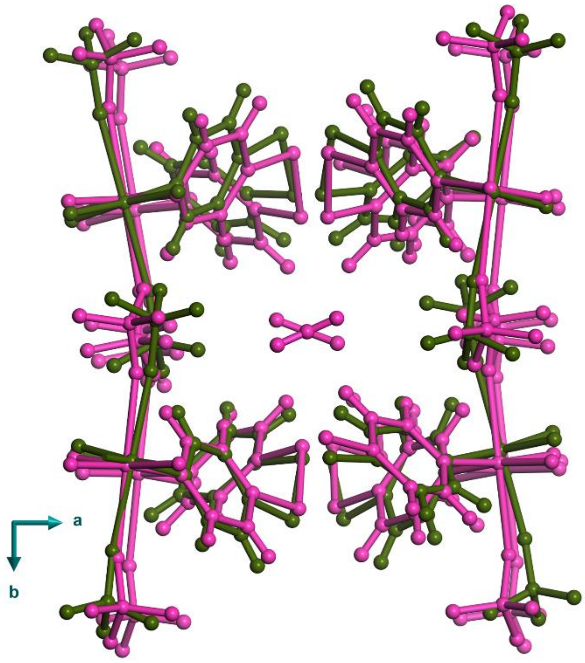

Supplementary Figure 9. The framework evolution of BFFOUR-Cu-dpds before and after activation. Dark green: BFFOUR-Cu-dpds; pink: BFFOUR-Cu-dpds@H_2O.

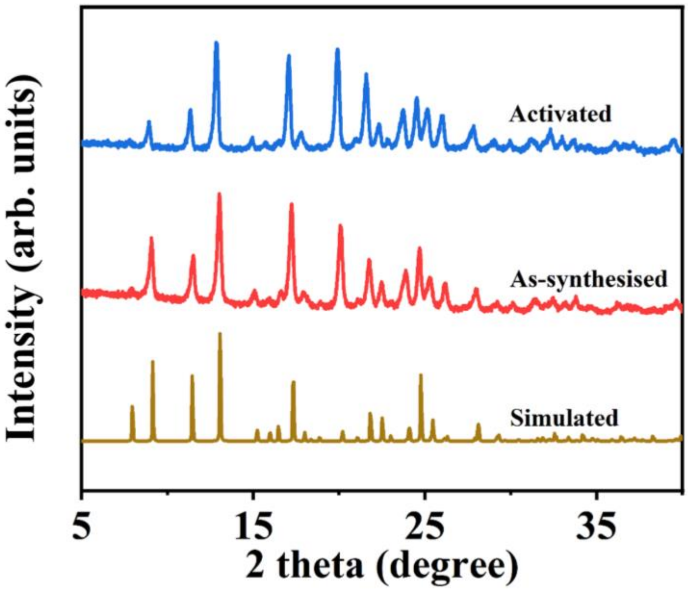

Supplementary Figure 10. Powder X-ray diffraction patterns. The powder X-ray diffraction patterns of BFFOUR-Cu-dpds.

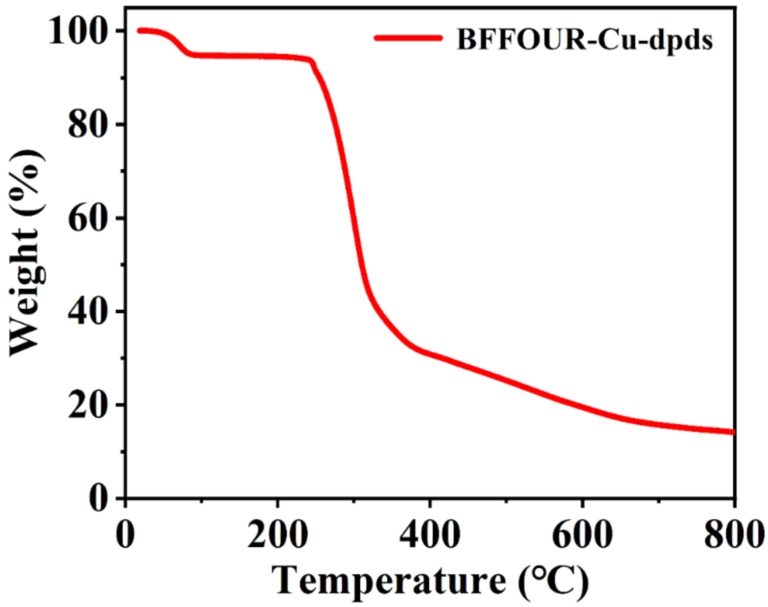

Supplementary Figure 11. TGA curve. The TGA curve of BFFOUR-Cu-dpds.

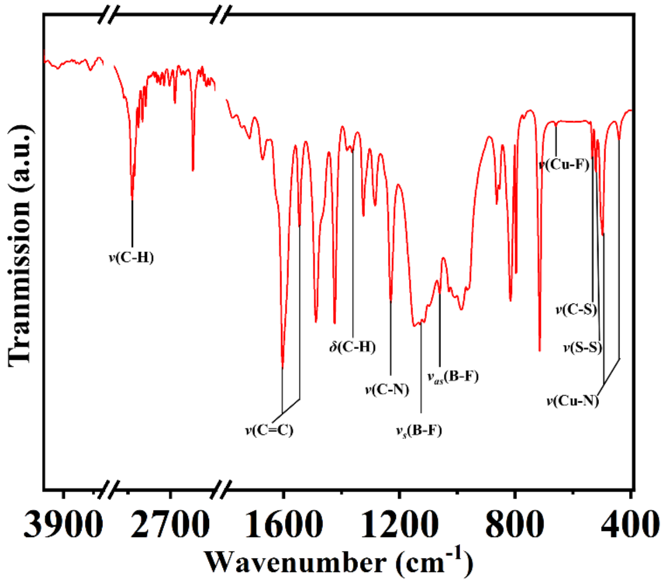

Supplementary Figure 12. FT-IR spectra of actived BFFOUR-Cu-dpds. Notation and acronym: *v* = stretching, *δ* =deformation, *s*= symmetric, *as*= asymmetric.[1-6]

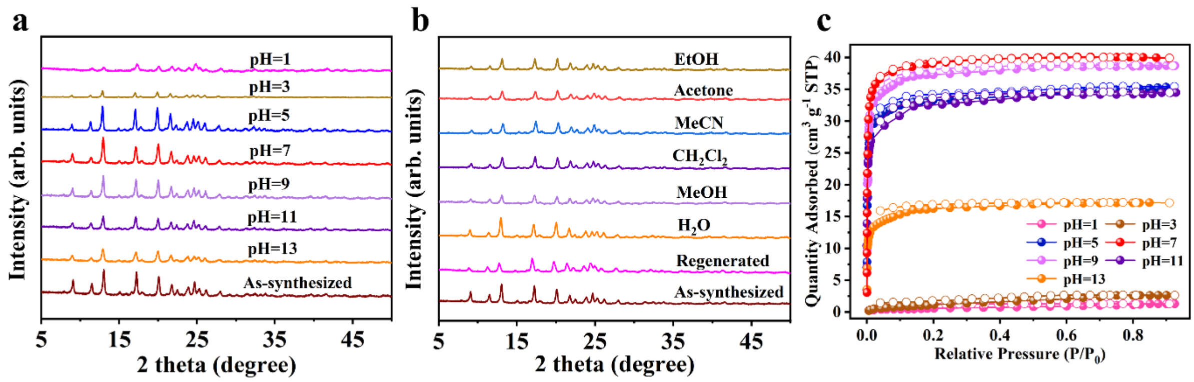

Supplementary Figure 13. PXRD patterns and CO_2 adsorption isotherms at 195 K after various treatments. PXRD patterns after various treatments in (a) water and organic solvents, (b) aqueous solutions at various pH values for 7 days, and (c) CO_2 adsorption isotherms at 195 K after immersing in various pH aqueous solutions for **7** days.

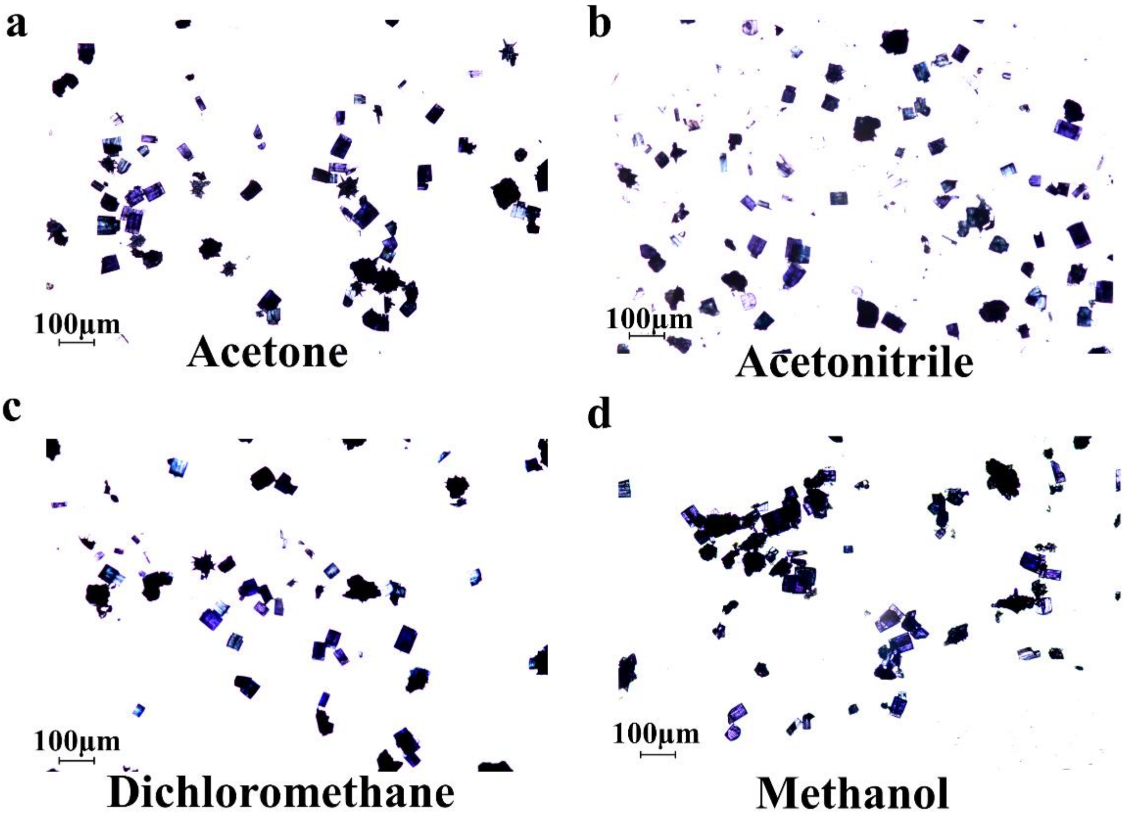

Supplementary Figure 14. Digital photographs of BFFOUR-Cu-dpds crystals after immersing in various organic solvents. Digital photographs of single-crystals BFFOUR-Cu-dpds after soaking in various organic solvents.

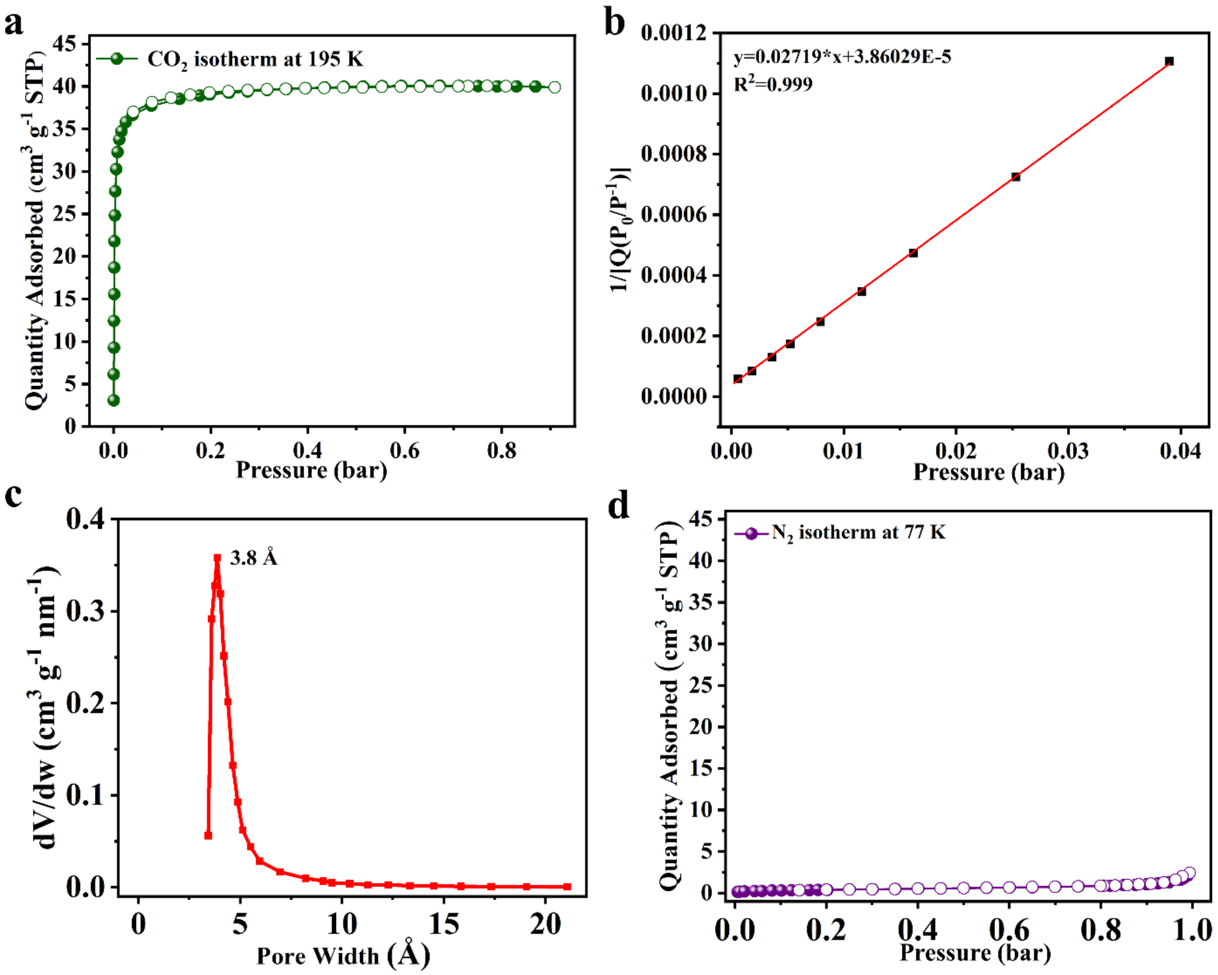

Supplementary Figure 15. Single-component adsorption isotherms of CO_2 (green) at 195 K and N_2 (purple) at 77 K. (a) CO_2 adsorption isotherm of BFFOUR-Cu-dpds at 195 K; (b) BET calculation plot; (c) Pore size distribution based on Horvath-Kawazoe model; (d) N_2 adsorption isotherm of BFFOUR-Cu-dpds at 77 K.

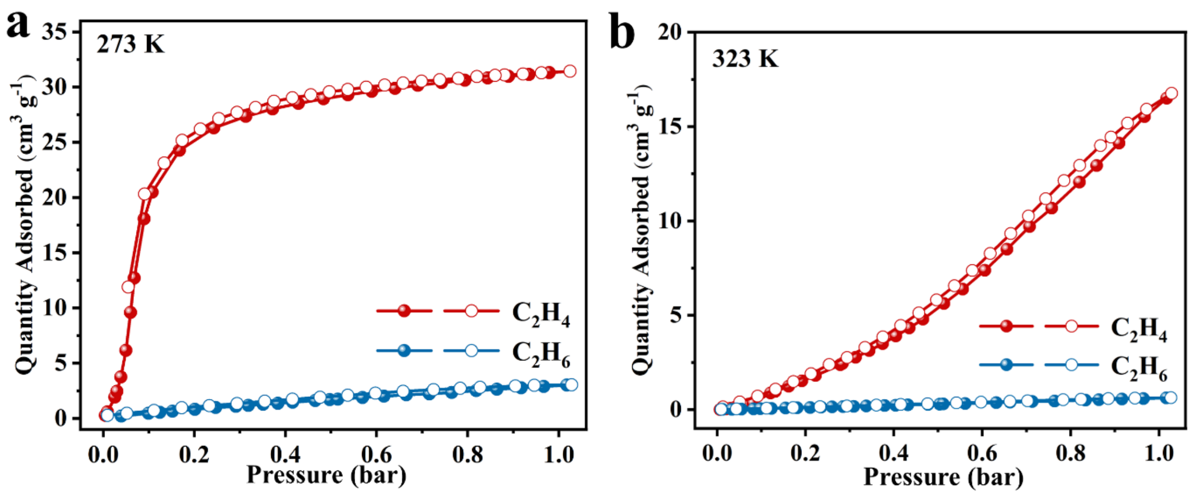

Supplementary Figure 16. Adsorption isotherms for C_2H_4 and C_2H_6 at 273 and 323 K. Adsorption isotherms of C_2H_4 and C_2H_6 on BFFOUR-Cu-dpds at (a) 273 and (b) 323 K.

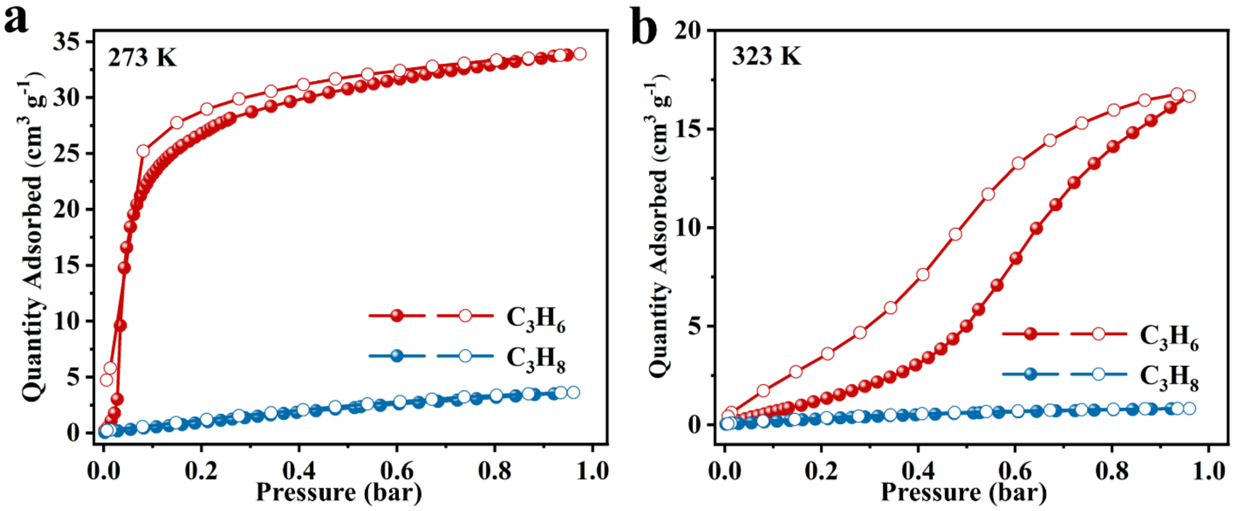

Supplementary Figure 17. Adsorption isotherms for C_3H_6 and C_3H_8 at 273 and 323 K. Adsorption isotherms of C_3H_6 and C_3H_8 on BFFOUR-Cu-dpds at (a) 273 and (b) 323 K.

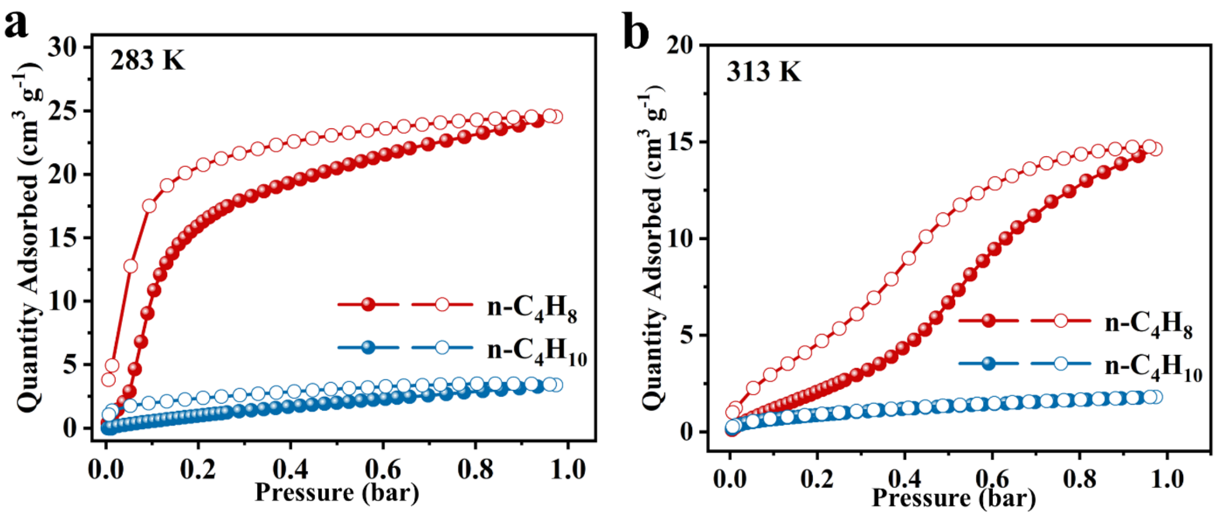

Supplementary Figure 18. Adsorption isotherms for n-C_4H_8 and n-C_4H_{10} at 283 and 313 K. Adsorption isotherms of n-C_4H_8 and n-C_4H_{10} on BFFOUR-Cu-dpds at (a) 283 and (b) 313 K.

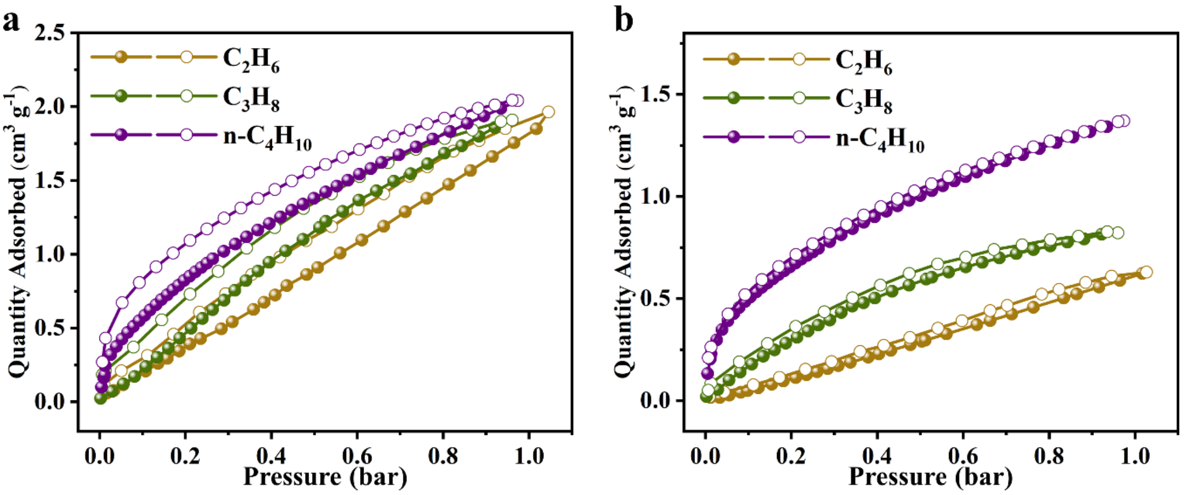

Supplementary Figure 19. Adsorption isotherms of C_2H_6, C_3H_8, and n-C_4H_{10} are at different temperatures. (a) Adsorption isotherms of C_2H_6, C_3H_8, and n-C_4H_{10} at 298 K; (b) adsorption isotherms of C_2H_6 and C_3H_8 at 323 K and n-C_4H_{10} at 313 K on BFFOUR-Cu-dpds.

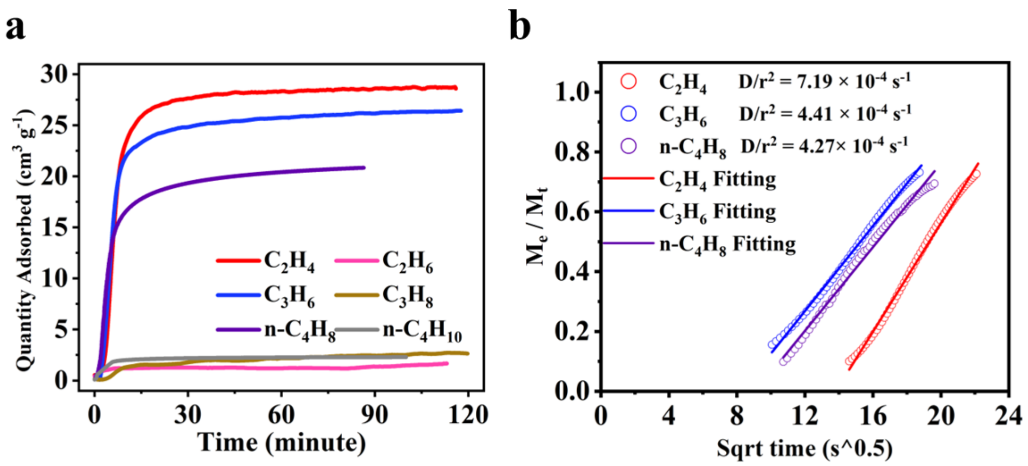

Supplementary Figure 20. **The kinetic adsorption and fitting curves** for **C_2-C_4 at 298 K and 1 bar.** (a) The kinetic adsorption curves and (b) fiting curves for C_2-C_4 olefins and paraffins at 1.0 bar.

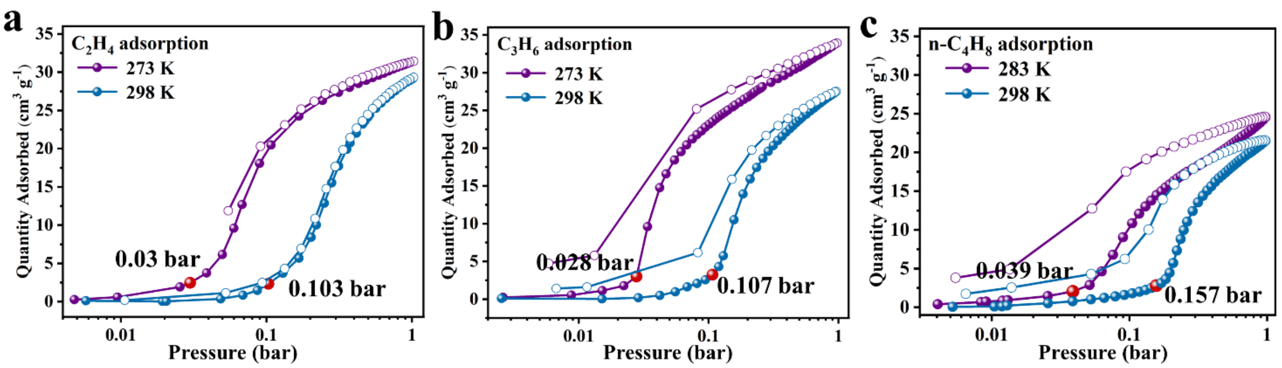

Supplementary Figure 21. The threshold pressure of C_2-C_4 at 273, 283, and 298 K. The threshold pressure for (a) C_2H_4 and (b) C_3H_6 at 273 K and 298 K, (c) n-C_4H_8 at 283 K and 298 K.

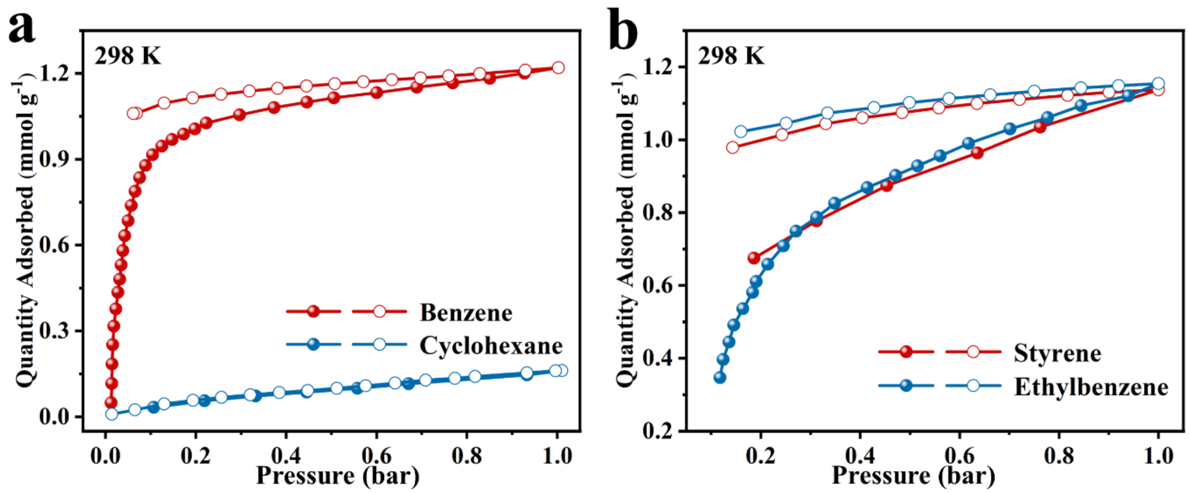

Supplementary Figure 22. Adsorption isotherms for benzene, cyclohexane, ethylbenzene, and ethylbenzene at 298 K. Adsorption isotherms for (a) benzene and cyclohexane and (b) styrene (ST) and ethylbenzene (EB) at 298 K on BFFOUR-Cu-dpds.

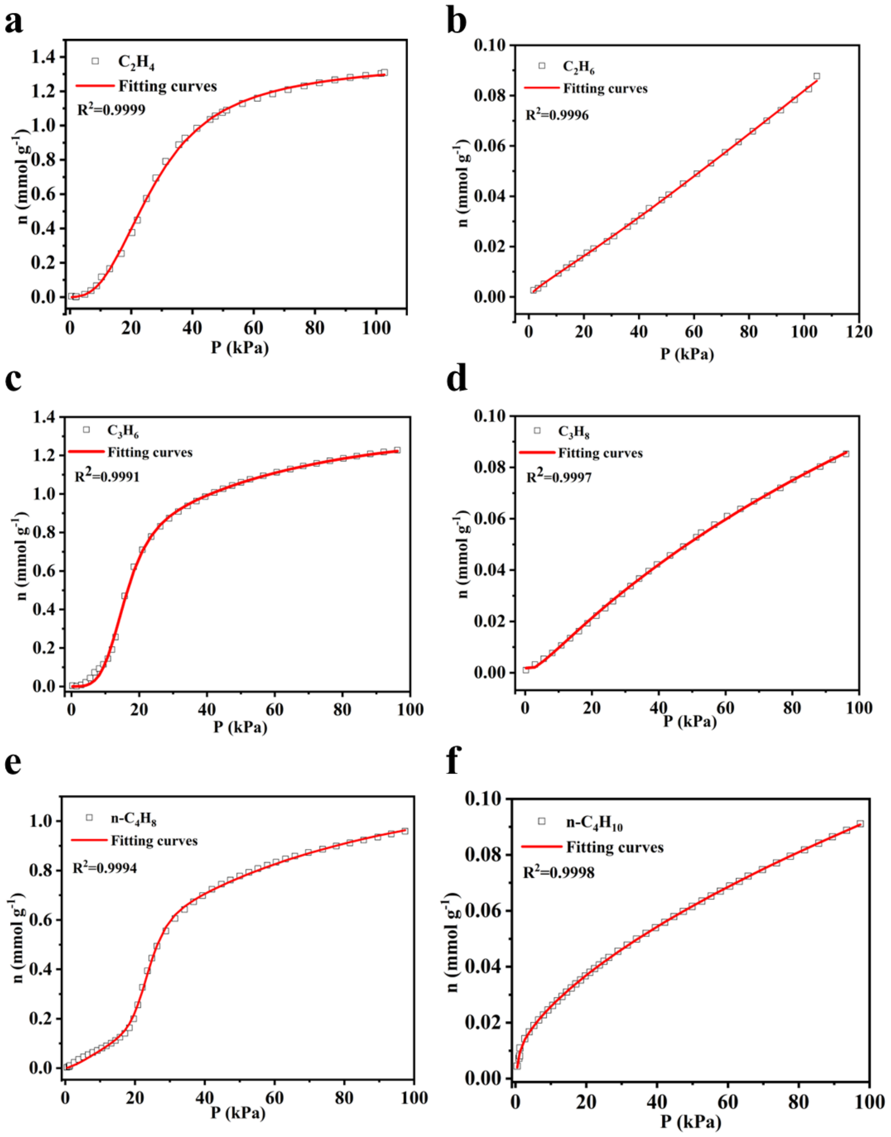

Supplementary Figure 23. DSLF fitting curves of adsorption isotherm for C_2-C_4 at 298 K. DSLF fitting curves for (a) C_2H_4 and (b) C_2H_6, (c) C_3H_6 and (d) C_3H_8, and (e) n-C_4H_8 and (f) n-C_4H_{10} adsorption isotherms at 298 K on BFFOUR-Cu-dpds.

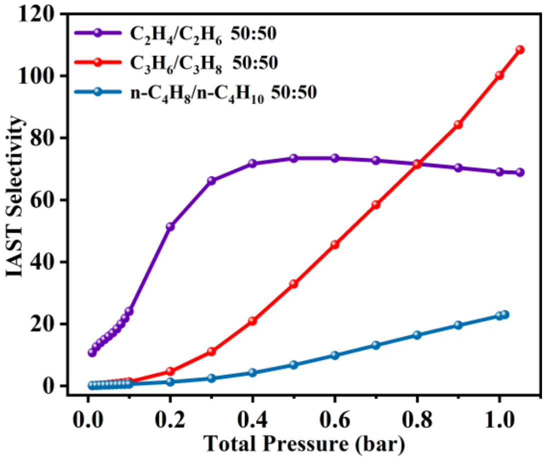

Supplementary Figure 24. The IAST selectivity of equimolar C_2H_4/C_2H_6, C_3H_6/C_3H_8, and n-C_4H_8/n-C_4H_{10}. IAST selectivity for C_2H_4/C_2H_6 (0.5/0.5, *v/v*) mixture, C_3H_6/C_3H_8 (0.5/0.5, *v/v*) mixture, and n-C_4H_8/n-C_4H_{10} (0.5/0.5, *v/v*) mixture on BFFOUR-Cu-dpds at 298K.

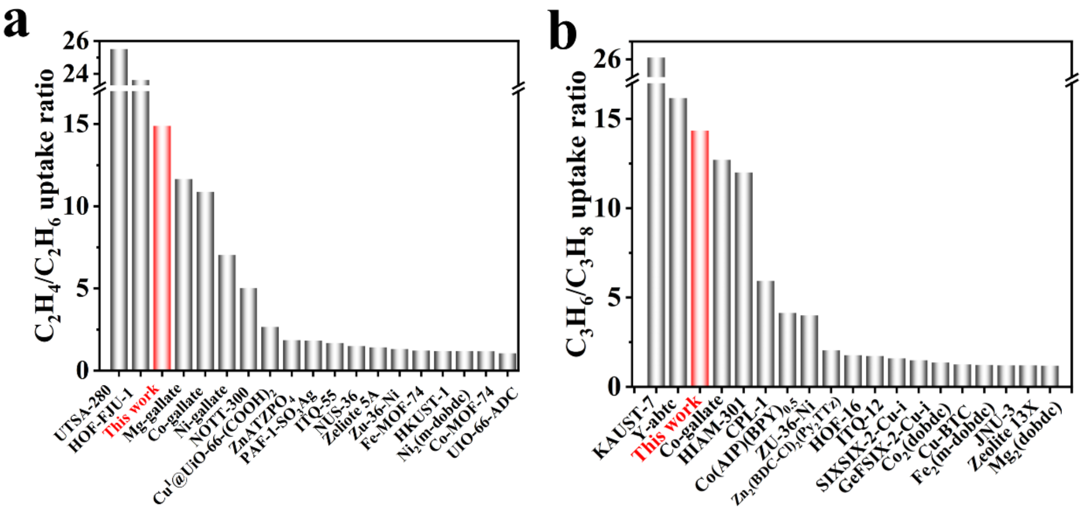

Supplementary Figure 25. Comparison of uptake ratio of C_2H_4/C_2H_6 and C_3H_6/C_3H_8. Comparison of (a) C_2H_4/C_2H_6 uptake ratio and (b) C_3H_6/C_3H_8 uptake ratio at 298 K and 1 bar with leading adsorbents. Ref [7-15, 17-27, 29-34]

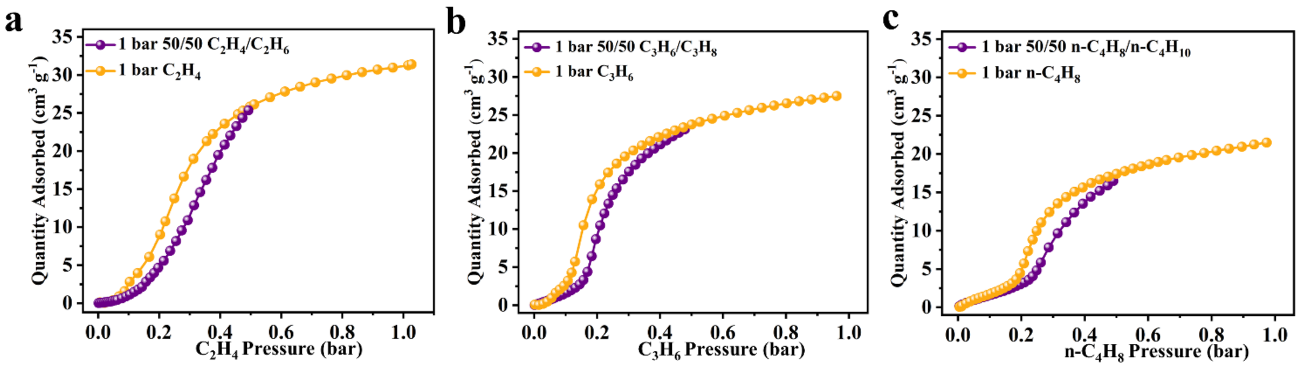

Supplementary Figure 26. Comparison of adsorption isotherms for pure olefin and equimolar olefin/paraffin gas-mixtures at 1.0 bar and 298 K. Adsorption isotherms of (a) pure C_2H_4 and equimolar C_2H_4/C_2H_6, (b) pure C_3H_6 and equimolar C_3H_6/C_3H_8, (c) pure n-C_4H_8 and equimolar n-C_4H_8/n-C_4H_{10} on BFFOUR-Cu-dpds.

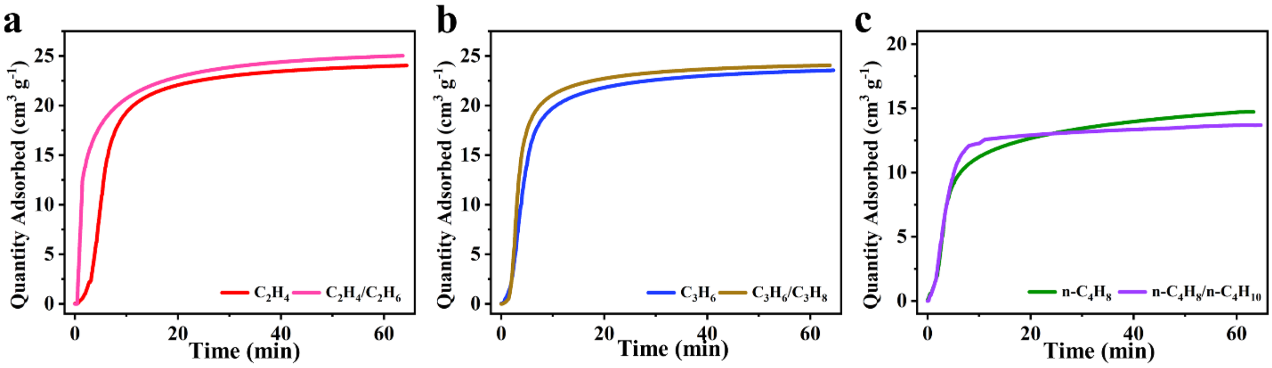

Supplementary Figure 27. Comparison of kinetic adsorption curves for pure olefins at 0.5 bar and equimolar olefin/paraffin gas-mixtures at 1.0 bar. Kinetic adsorption curve of (a) pure C_2H_4 and equimolar C_2H_4/C_2H_6, (b) pure C_3H_6 and equimolar C_3H_6/C_3H_8, (c) pure n-C_4H_8 and equimolar n-C_4H_8/n-C_4H_{10} on BFFOUR-Cu-dpds.

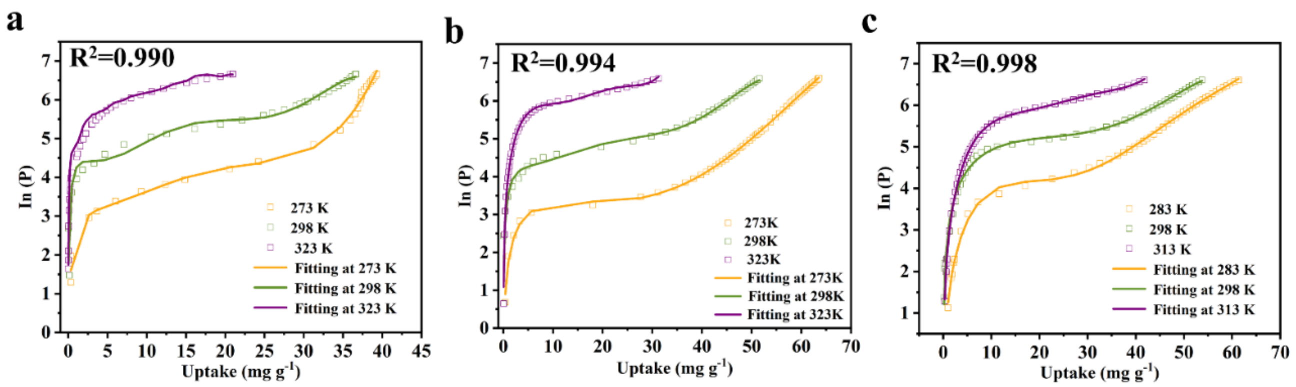

Supplementary Figure 28. Virial curves fitting for C_2H_4, C_3H_6 and n-C_4H_8 isotherms. Virial equation fitting of (a) C_2H_4, (b) C_3H_6, and (c) n-C_4H_8 adsorption isotherms on BFFOUR-Cu-dpds at different temperatures.

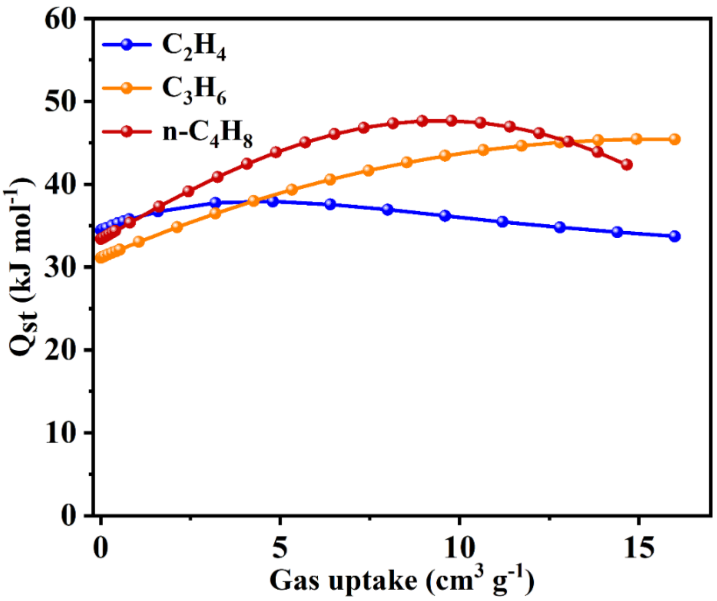

Supplementary Figure 29. The adsorption heat for C_2H_4, C_3H_6, and n-C_4H_8. The calculated Q_{st} for C_2H_4, C_3H_6, and n-C_4H_8 on BFFOUR-Cu-dpds.

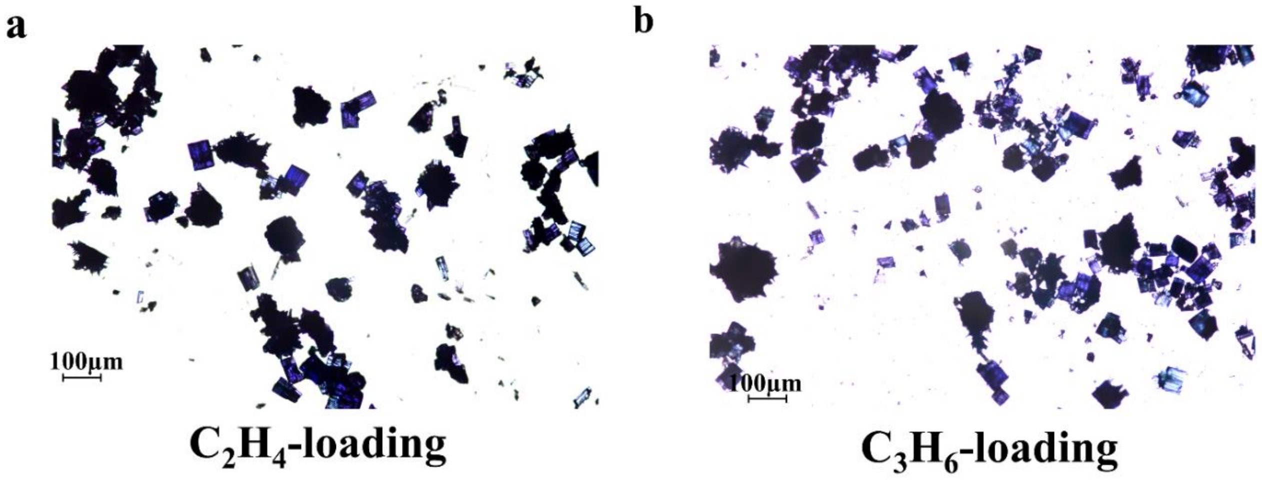

Supplementary Figure 30. Digital photographs of BFFOUR-Cu-dpds crystals after gas-loading.

Digit photos of single-crystal BFFOUR-Cu-dpds after (a) C_2H_4- and (b) C_3H_6-loading.

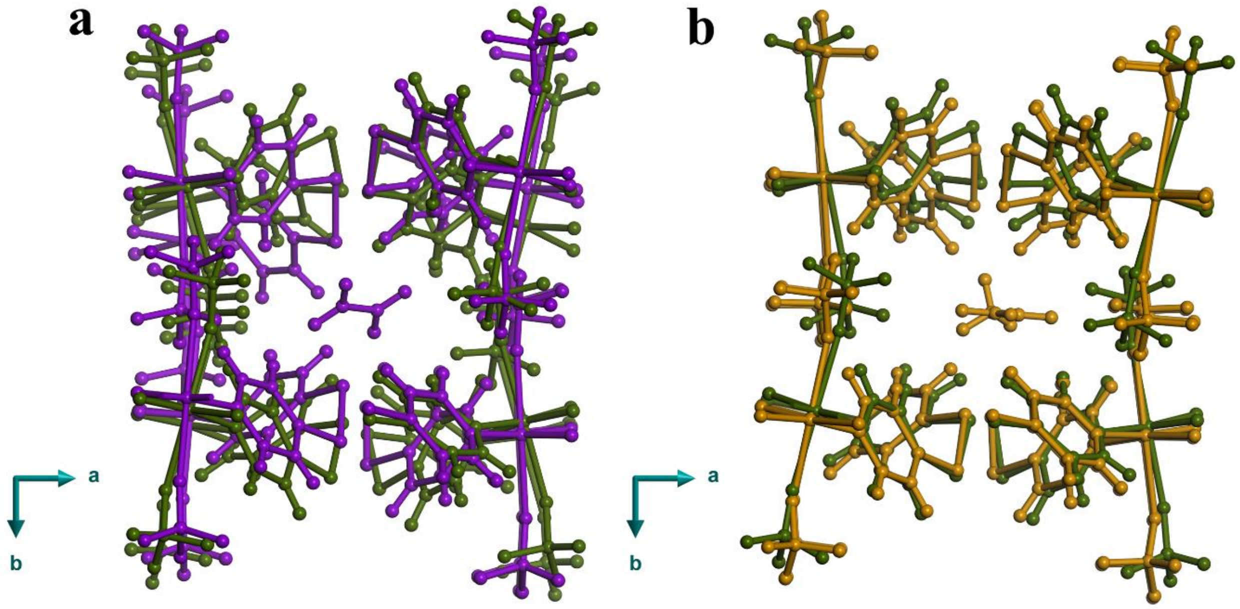

Supplementary Figure 31. Comparison of the C_2H_4-loaded structure and C_3H_6-loaded structures with the activated structure. The comparison of the (a) C_2H_4-loading structure and (b) C_3H_6-loading structure with the activated structure.

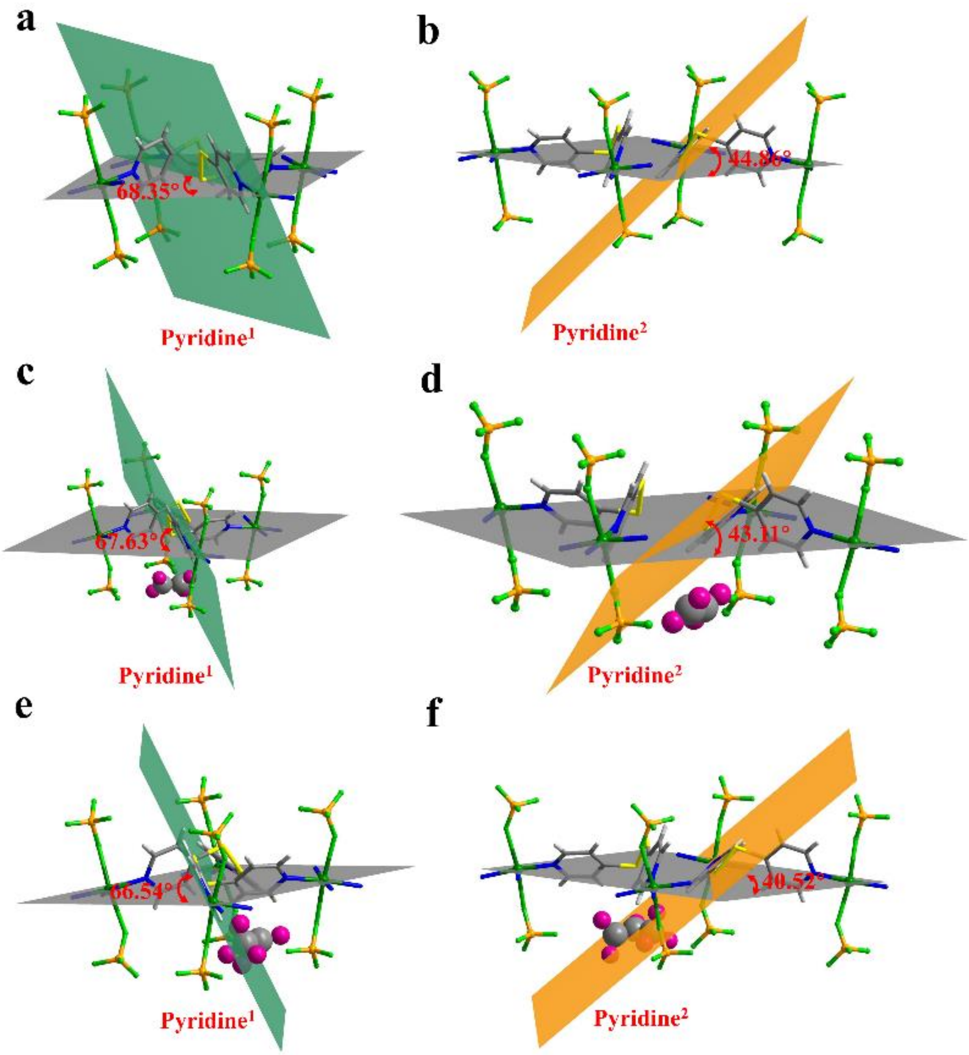

Supplementary Figure 32. Geometry optimizations of gas-loaded BFFOUR-Cu-dpds. The dihedral angle between the pyridine plane and the Cu^{2+} plane in (a, b) activated BFFOUR-Cu-dpds, (c, d) C_2H_4-loaded BFFOUR-Cu-dpds, and (e, f) C_3H_6-loaded BFFOUR-Cu-dpds.

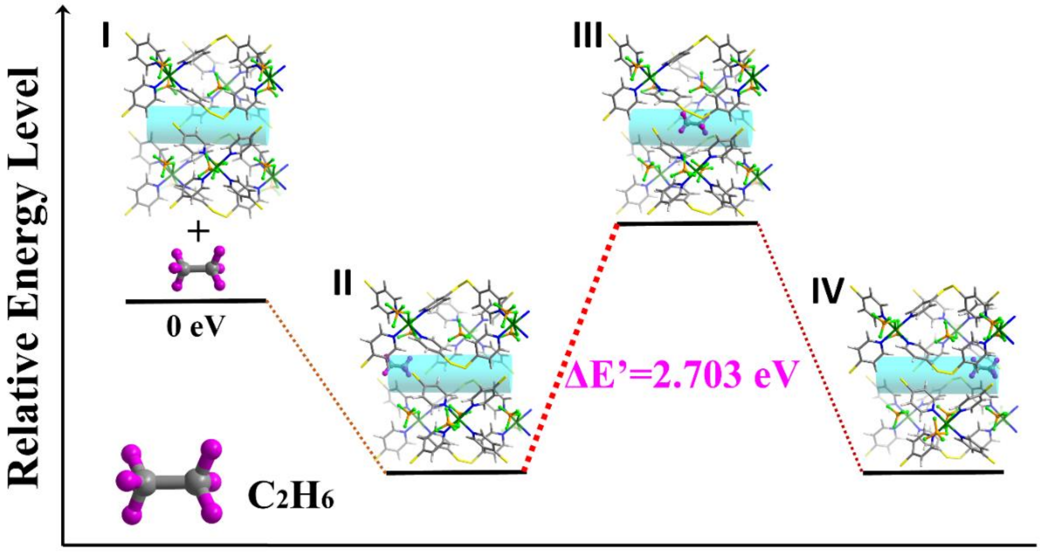

Supplementary Figure 33. The intermediate state energy for C_2H_6 diffusion. The energy pathway and corresponding energy levels for C_2H_6 for entering BFFOUR-Cu-dpds.

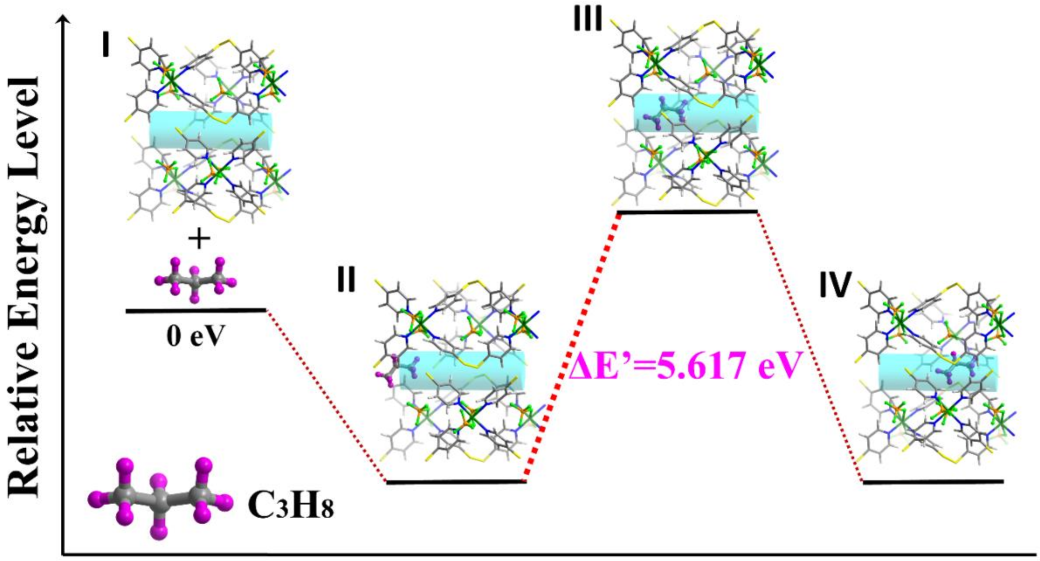

Supplementary Figure 34. The intermediate state energy for C_3H_8 diffusion. The energy pathway and corresponding energy levels for C_3H_8 for entering BFFOUR-Cu-dpds.

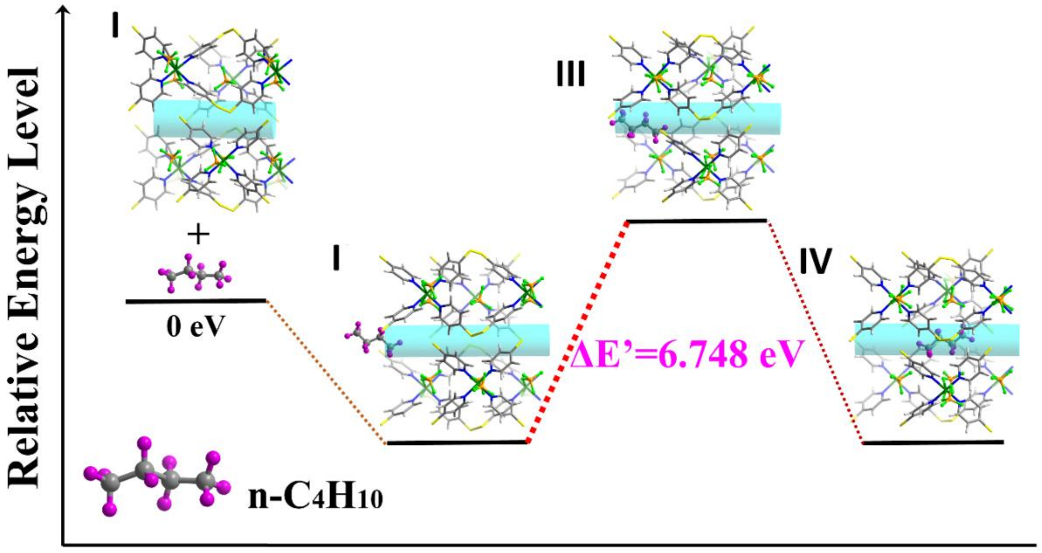

Supplementary Figure 35. The intermediate state energy for n-C_4H_{10} diffusion. The energy pathway and corresponding energy levels for n-C_4H_{10} for entering BFFOUR-Cu-dpds.

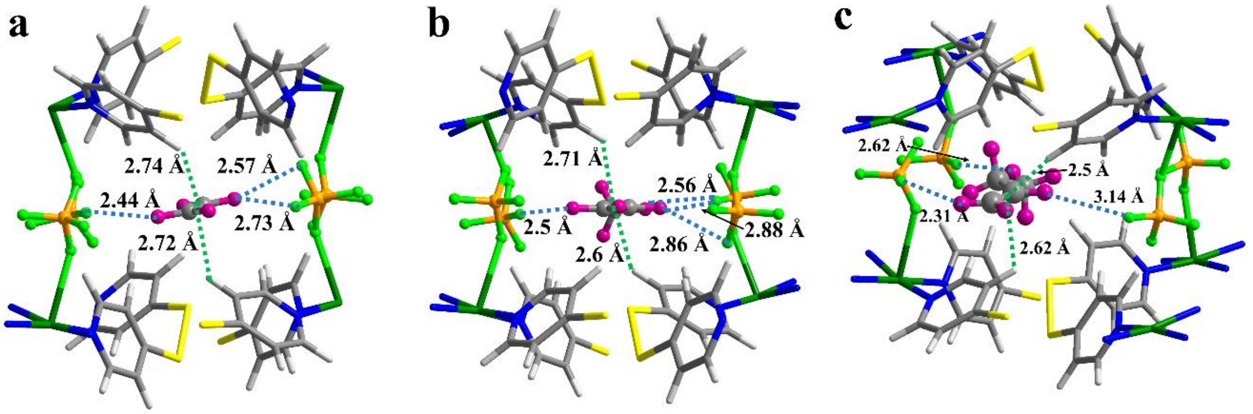

Supplementary Figure 36. DFT-D calculated host-guest interactions. (a) C_2H_4-loaded, (b) C_3H_6-loaded, and (c) n-C_4H_8-loaded framework of BFFOUR-Cu-dpds along the *c* axis. Color code: F, green; Cu, dark green; C, gray; H (in framework), light gray; H (in hydrocarbons), dark purple; N, blue. S, yellow; blue line: C-H···F interaction; green line: C-H···π (olefin) binding.

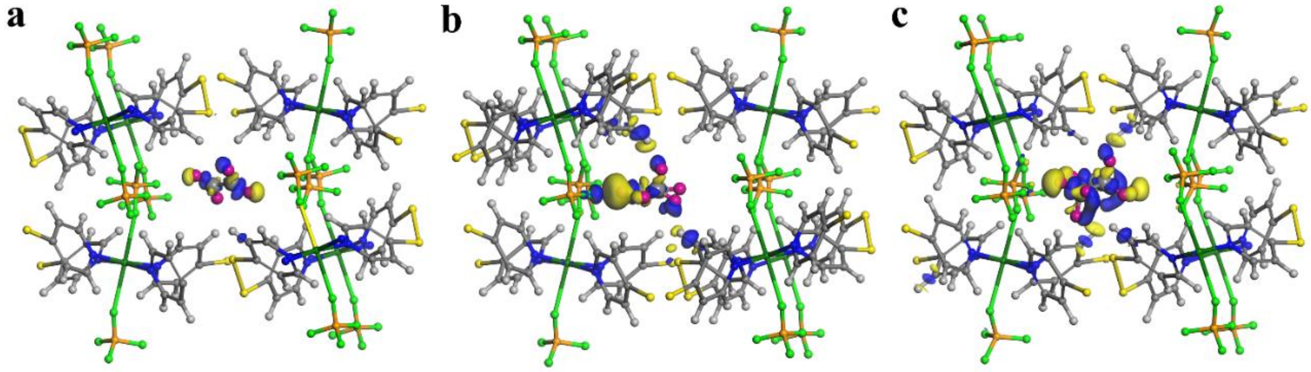

Supplementary Figure 37. Charge density bias maps of C_2H_4, C_3H_6 and n-C_4H_8. Charge density bias plots showing the interactions between (a) C_2H_4, (b) C_3H_6, (c) n-C_4H_8 and the framework. Color code: F, green; Cu, dark green; C, gray; H (in framework), light gray; H (in hydrocarbons), dark purple; N, blue. S, yellow. Charge distribution: positive charge, yellow; negative charge, blue.

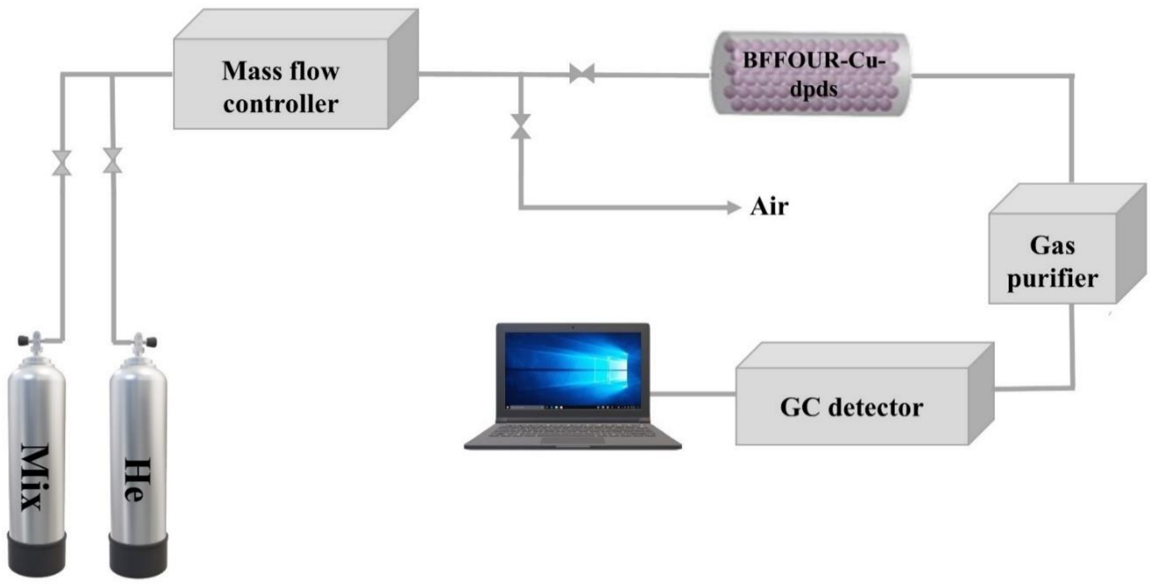

Supplementary Figure 38. Diagram of the breakthrough experiment. Schematic illustration of the setup for breakthrough experiments.

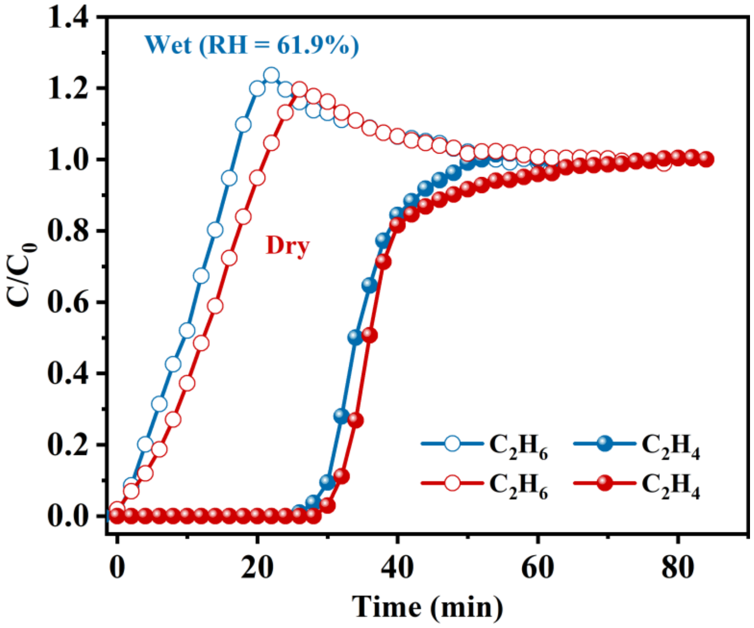

Supplementary Figure 39. Breakthrough curves for C_2H_4/C_2H_6 in humid and dry conditions. Breakthrough curves for C_2H_4/C_2H_6 (0.5/0.5, *v/v*, 1.0 ml min^{-1} and 298 K) mixture in dry and humid conditions.

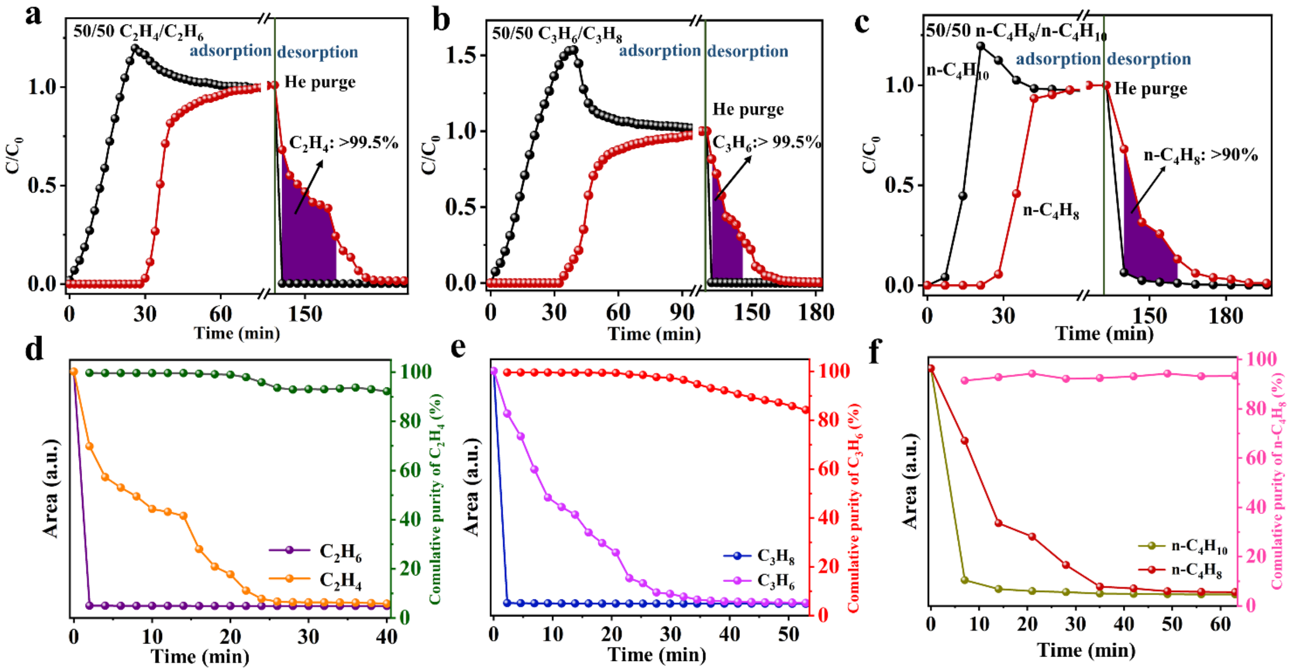

Supplementary Figure 40. Breakthrough curves and regeneration curves of C_2-C_4 at 298 K. The breakthrough curve of (a) C_2H_4/C_2H_6 (0.5/0.5, *v/v*), (b) C_3H_6/C_3H_8 (0.5/0.5, *v/v*), and (c) n-C_4H_8/n-C_4H_{10} (0.5/0.5, *v/v*) at 298 K; and desorption curve of (d) C_2H_4/C_2H_6 (0.5/0.5, *v/v*), (f) C_3H_6/C_3H_8 (0.5/0.5, *v/v*), and (f) n-C_4H_8/n-C_4H_{10} (0.5/0.5, *v/v*). The signals of desorption conditions: 5 ml min^{-1} He and 333 K for 12 h.

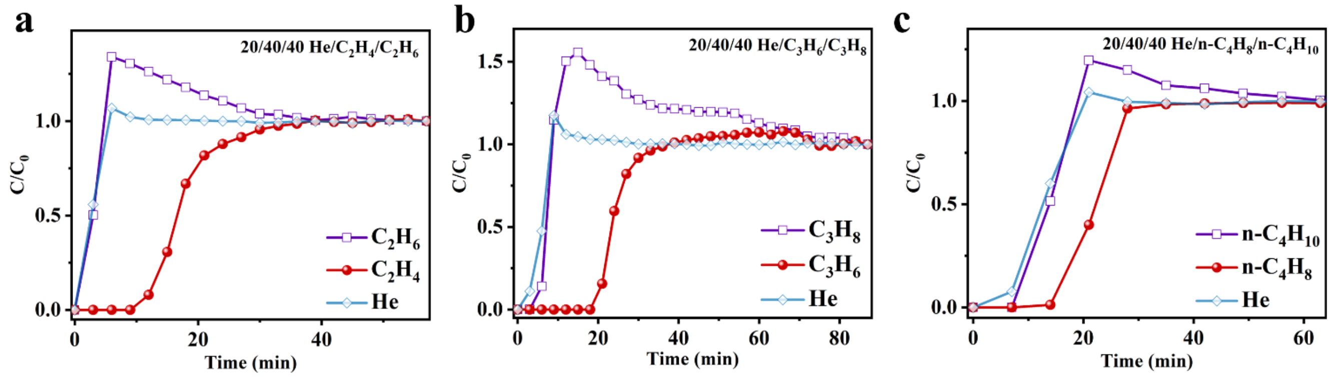

Supplementary Figure 41. Breakthrough curves for He/olefin/paraffin (0.2/0.4/0.4, *v/v/v*, 2.5 ml min^{-1} and 298 K) mixture. Breakthrough curves for (a) He/C_2H_4/C_2H_6 (0.2/0.4/0.4, *v/v/v*) mixture, (b) He/C_3H_6/C_3H_8 (0.2/0.4/0.4, *v/v/v*) mixture, and (c) He/n-C_4H_8/n-C_4H_{10} (0.2/0.4/0.4, *v/v/v*) mixture.

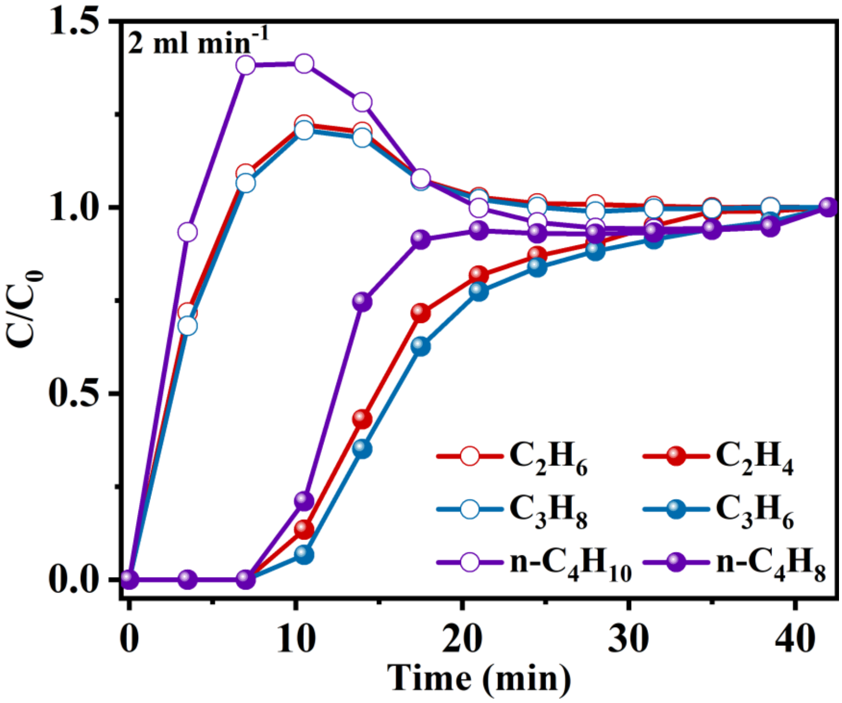

Supplementary Figure 42. The breakthrough curve for equimolar of C_2H_4, C_2H_6, C_3H_6, C_3H_8, n-C_4H_8, and n-C_4H_{10}. The adsorption breakthrough curve for C_2H_4/C_2H_6/C_3H_6/C_3H_8/n-C_4H_8/n-C_4H_{10} (0.167/0.167/0.167/0.167/0.167/0.167, *v/v/v/v/v/v*) on BFFOUR-Cu-dpds at 2 ml min^{-1} and 298 K.

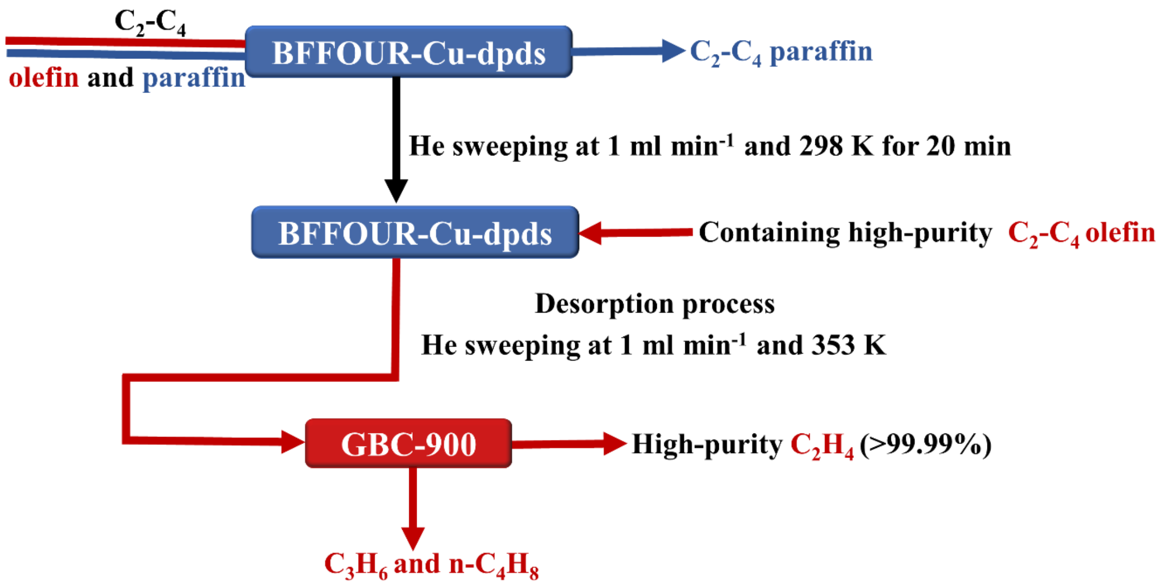

Supplementary Figure 43. C_2-C_4 six-component purification process diagram. The process flow for direct C_2H_4 production from six-component gas-mixtures of C_2-C_4 olefins and paraffins.

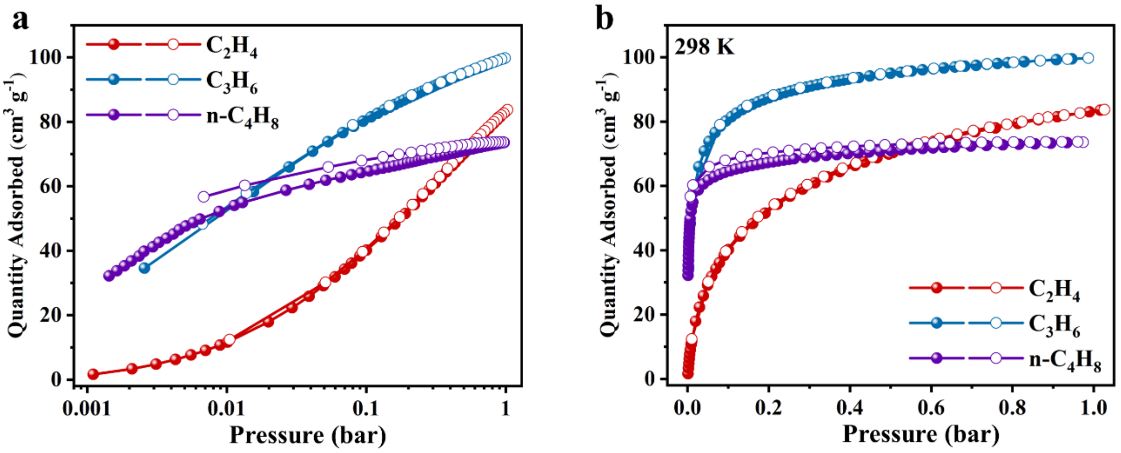

Supplementary Figure 44. Adsorption isotherms of GBC-900 for C_2H_4, C_3H_6, and n-C_4H_8 at 298 K. Adsorption isotherms of C_2H_4, C_3H_6, and n-C_4H_8 on GBC-900 at 298 K: (a) logarithmic model and (b) linear model.

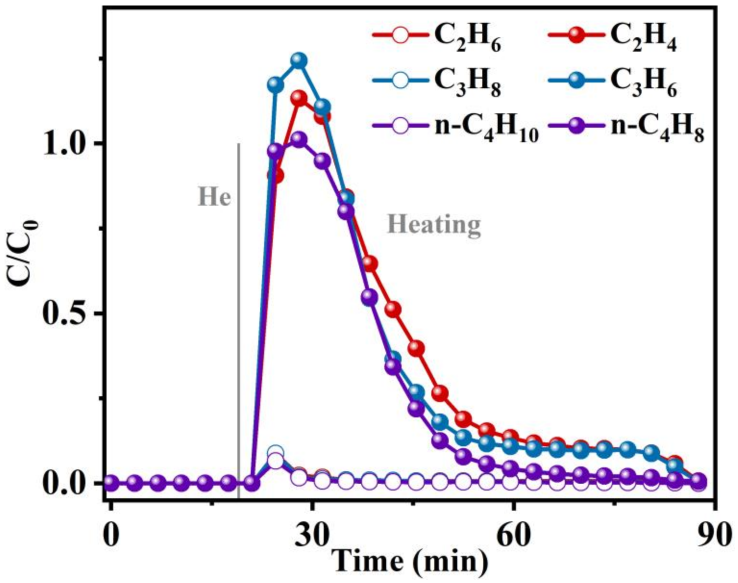

Supplementary Figure 45. C_2-C_4 six-component gas desorption curve. The desorption curve of trapped olefin mixture in BFFOUR-Cu-dpds with 1.0 ml min^{-1} He sweeping at 353 K.

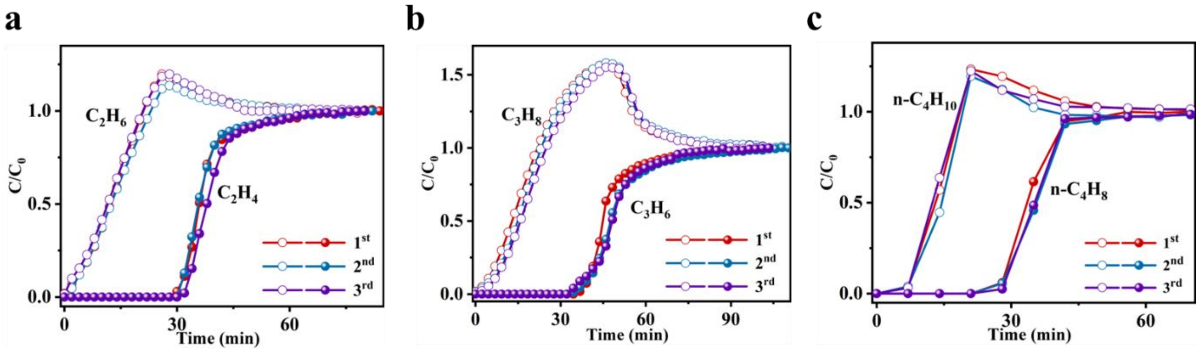

Supplementary Figure 46. Cyclic breakthrough curves for C_2H_4/C_2H_6, C_3H_6/C_3H_8 and n-C_4H_8/n-C_4H_{10}. Three breakthrough cycles curves of (a) C_2H_4/C_2H_6 (0.5/0.5, *v/v*), (b) C_3H_6/C_3H_8 (0.5/0.5, *v/v*), and (c) n-C_4H_8/n-C_4H_{10} (0.5/0.5, *v/v*) on BFFOUR-Cu-dpds at 1 ml min^{-1} and 298 K. The signals of desorption conditions: 5 ml min^{-1} He and 333 K for 90 min.

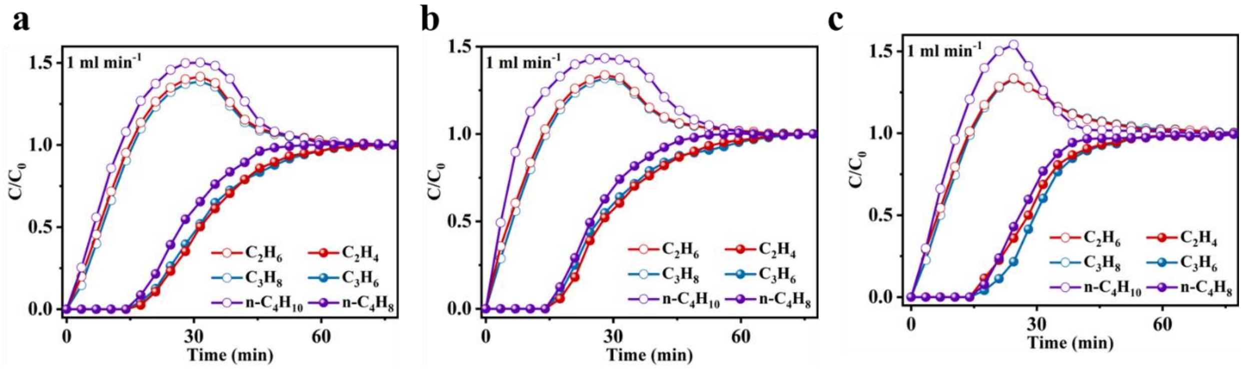

Supplementary Figure 47. Cyclic breakthrough curves for equimolar C_2H_4, C_2H_6, C_3H_6, C_3H_8, n-C_4H_8, and n-C_4H_{10} at flow rate of 1.0 ml min^{-1}. Three breakthrough cycles for C_2H_4/C_2H_6/C_3H_6/C_3H_8/n-C_4H_8/n-C_4H_{10} (0.167/0.167/0.167/0.167/0.167/0.167, *v/v/v/v/v/v*) on BFFOUR-Cu-dpds at 1.0 ml min^{-1} and 298 K. The signals of desorption conditions: 5 ml min^{-1} He and 333 K for 90 min.

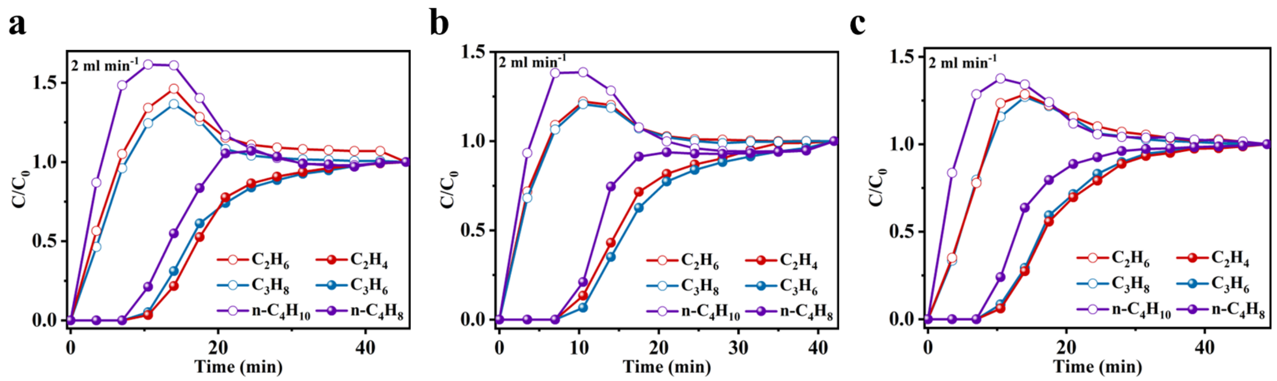

Supplementary Figure 48. Cyclic breakthrough curves for equimolar C_2H_4, C_2H_6, C_3H_6, C_3H_8, n-C_4H_8, and n-C_4H_{10} at flow rate of 2.0 ml min^{-1}. Three breakthrough cycles for C_2H_4/C_2H_6/C_3H_6/C_3H_8/n-C_4H_8/n-C_4H_{10} (0.167/0.167/0.167/0.167/0.167/0.167, *v/v/v/v/v/v*) on BFFOUR-Cu-dpds at 2.0 ml min^{-1} and 298 K.The signals of desorption conditions: 5 ml min^{-1} He and 333 K for 90 min.

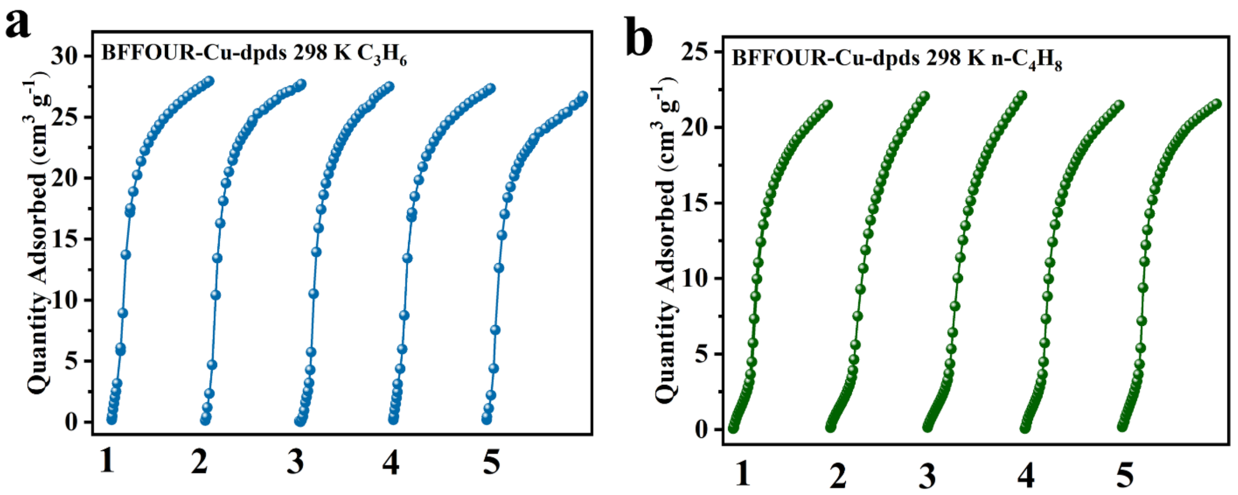

Supplementary Figure 49. Cyclic adsorption isotherms of C_3H_6 and n-C_4H_8 at 298 K. Five repeated adsorption isotherms for (a) C_3H_6 and (b) n-C_4H_8 on BFFOUR-Cu-dpds at 298 K and 1 bar. The signals of desorption conditions: vacuum 353 K for 12 h.

Supplementary Table S1. Crystal data of as-synthesized BFFOUR-Cu-dpds, activated BFFOUR-Cu-dpds, C_3H_6@BFFOUR-Cu-dpds, and C_2H_4@BFFOUR-Cu-dpds.

Complex	As-synthesized BFFOUR-Cu-dpds	Activated BFFOUR-Cu-dpds	C_3H_6@BFFOUR-Cu-dpds	C_2H_4@BFFOUR-Cu-dpds
Formula.	$C_{20}H_{20}B_2CuF_8N_4O_2S_4$[+$H_2O$]	$C_{20}H_{16}B_2CuF_8N_4S_4$	$C_{21.5}H_{19}B_2Cu_1F_8N_4S_4$[+$C_3H_6$]	$C_{20.75}H_{17.5}B_2CuF_8N_4S_4$[+$C_2H_4$]
F.W.	713.8	677.78	698.81	688.3
Crystal size (mm)	0.12×0.1×0.1	0.1×0.1×0.1	0.15×0.12×0.1	0.15×0.12×0.1
Crystal system	Orthorhombic	Orthorhombic	Orthorhombic	Orthorhombic
Space group	*Ccc2*	*Ccc2*	*Ccc2*	*Ccc2*
a /Å	13.576(4)	13.546 (7)	13.458 (4)	13.3959(9)
b /Å	19.393(6)	19.317 (4)	19.369 (6)	19.2894(9)
c /Å	10.716(4)	10.7628 (13)	10.747 (3)	10.7599(5)
α/°	90	90	90	90
β/°	90	90	90	90
γ/°	90	90	90	90
volume/Å3	2821.2(16)	2816.3 (16)	2801.5 (15)	2780.3 (3)
Z	4	4	4	4
D_c (g cm^{-3})	1.681	1.599	1.606	1.644
μ / mm^{-1}	6.495	1.144	5.89	6.507
θ range (°)	6.914-121.514	7.348-58.246	6.985-120.108	6.99-108.228
F000	1436	1356	1404	1380
R_1 [$I \geq 2\sigma(I)$] [a]	0.0414	0.0843	0.0996	0.0592
ωR_2(all data)[b]	0.1137	0.2738	0.3089	0.1599
GOF	1.064	1.058	1.090	1.055
CCDC	2162794	2162081	2162073	2162071

Supplementary Table S2. The fitting parameters of the dual-site Langmuir-Freundlich equation model for C_2H_4, C_2H_6, C_3H_6, C_3H_8, n-C_4H_8, and n-C_4H_{10} adsorption on BFFOUR-Cu-dpds. Dual-site Langmuir-Freundlich (DSLF) model is listed below:

$$N = N_1^{\max} \times \frac{b_1 P^{c_1}}{1+b_1 P^{c_1}} + N_2^{\max} \times \frac{b_2 P^{c_2}}{1+b_2 P^{c_2}}$$

Adsorbates	N_1^{max} (mmol g^{-1})	b_1 (bar^{-1})	C_1	N_2^{max} (mmol g^{-1})	b_2 (bar^{-1})	C_2
C_2H_4 (298 K)	0.7686466	0.000192303	4.451723	0.7575	0.041505	1.032607
C_2H_6 (298 K)	2.266979	0.000687412	0.8198868	2.266979	0.000687412	0.8198868
C_3H_6 (298 K)	0.8419861	9.5289E-6	4.193398	0.5079988	2.427431E-4	2.064262
C_3H_8 (298 K)	0.0067056	1.217249	0.4009188	0.3302159	0.003252615	1.227404
n-C_4H_8 (298 K)	0.87337	3.95E-3	1.35399	0.38638	1.41627E-12	9.38565
n-C_4H_{10} (298 K)	1.55635	2.78E-3	0.66162	6.15E-3	1.46165	2.34353

Supplementary Table S3. The virial parameters for calculated Q_{st} of C_2H_4 and C_3H_6 on BFFOUR-Cu-dpds at 273 K, 298 K, and 323 K up to 1.0 bar, and n-C_4H_8 on BFFOUR-Cu-dpds at 283 K, 298 K, and 313 K up to 1.0 bar.

Viral Coefficient	C_2H_4 Value	C_3H_6 Value	n-C_4H_8 Value
a_0	-4141.169	-3742.398	-4013.387
a_1	-185.1459	-120.1343	-120.43
a_2	26.48480	2.168967	0.1950658
a_3	-1.4000193	-6.048386E-3	0.1070342
a_4	3.305556E-2	1.661253E-4	-1.420702E-3
a_5	-2.78237E-4	-1.595323E-6	6.542909E-6
b_0	18.61167	15.84738	15.85082
b_1	-9.637695E-2	0.2930295	0.4978253
b_2	-1.23184E-3	-5.103154E-3	-1.12888E-2
Equation	$y = Ln(x)+1/T(a_0+a_1x+a_2x^2+a_3x^3+a_4x^4+a_5x^5) + (b_0+b_1x+b_2x^2)$		

Supplementary TableS4. Comparison of the adsorption capacity and C_2H_4/C_2H_6 (0.5/0.5, *v/v*) selectivity and heat of adsorption data of BFFOUR-Cu-dpds with other best-performing materials.

Sample	Measurement Conditions	S_{BET} (m^2 g^{-1})	C_2H_4 (cm^3 g^{-1})	C_2H_6 (cm^3 g^{-1})	Q_{st} (kJ mol^{-1}) C_2H_4	C_2H_4/C_2H_6 uptake ratio	C_2H_4/C_2H_6 (50/50) IAST selectivity	Ref.
BFFOUR-Cu-dpds	1 bar, 298 K	140	29.34	1.97	34.44	14.88	68.8	**This work**
Ni-gallate	1 bar, 298 K	424	44.13	6.27	32	7.03	16.8	[7]
Co-gallate	1 bar, 298 K	475	75.49	6.94	44	10.87	52	[7]
Mg-gallate	1 bar, 298 K	559	67.87	5.82	39	11.65	37.3	[7]
NOTT-300	1 bar, 298 K	1370	95.87	19.04	16	5.03	48.7	[8]
UTSA-280	1 bar, 298 K	331	56	2.2	34.1	25.51	>10000	[9]
HOF-HJU-1	1bar, 333 K	385	38.75	1.64	33	23.63	64	[10]
Cu^{I}@UiO-66 -$(COOH)_2$	1 bar, 298 K	320	41.66	15.68	48.5-	2.66	80.8	[11]
Zeolite 5A	1 bar, 298 K	457	54.88	38.53	37	1.42	4.5	[12]
Co-MOF-74	1 bar, 298 K	1341	139.10	117.6	43.6	1.18	5.82	[13]
Fe-MOF-74	1 bar, 298 K	1295	136.64	112	55	1.22	13.6	[13]
$ZnAtzPO_4$	1 bar, 298 K	-	43.0	23.07	17	1.85	12.4	[14]
UIO-66-ADC	1 bar, 298 K	556	38.08	35.84	36	1.06	1.8	[15]
PAF-1-SO_3Ag	1 bar, 298 K	783	90.94	49.95	106	1.82	26.9	[16]

NUS-36	1 bar, 298 K	79	33.6	22.4	44	1.5	4.1	[15]
NaETS-10	1 bar, 298 K	289	38.08	29.12	-	1.3	14.7	[17]
HKUST-1	1 bar, 298 K	1500	161.28	135.07	39	1.19	3.6	[18]
Ni_2(m-dobdc)	1 bar, 298 K	-	152.32	127.68	55	1.19	15.1	[19]
Co_2(m-dobdc)	1 bar, 298 K	-	159.04	132.16	47	1.2	15.2	[19]
ZU-36-Ni	1 bar, 298 K	246	44.8	34.05	26	1.32	3.2	[20]
ITQ-55	1 bar, 298 K	-	28.67	17.02	-	1.68	90	[21]

Supplementary Table S5. Comparison of the adsorption capacity and C_3H_6/C_3H_8 (0.5/0.5, *v/v*) selectivity and heat of adsorption data of BFFOUR-Cu-dpds with other best-performing materials.

Sample	Measurement Conditions	S_{BET} (m^2 g^{-1})	C_3H_6 (cm^3 g^{-1})	C_3H_8 (cm^3 g^{-1})	Q_{st} (kJ mol^{-1}) C_3H_6	C_3H_6/C_3H_8 (50/50) uptake ratio	C_3H_6/C_3H_8 (50/50) IAST selectivity	Ref.
BFFOUR-Cu-dpds	1 bar, 298 K	140	27.33	1.90	31.13	14.35	108.4	**This work**
Co-gallate	1 bar, 298 K	487	39.87	3.14	41	12.71	333	[22]
KAUST-7	1 bar, 298 K	280	31.58	1.21	57.4	26.11	-	[23]
Y-abtc	1 bar, 298 K	427	43.46	2.69	50	16.17	-	[24]
HIAM-301	1 bar, 298 K	579	70.78	<6.72	~27	11.47	>150	[25]
JNU-3	1 bar, 303 K	588	58.24	48.83	39.2	1.21	513	[26]
$Zn_2(BDC\text{-}Cl)_2(Py_2TTZ)$	1 bar, 273 K	441	74.4	36.2	2.5	2..05	5.2	[27]
$Co(AIP)(BPY)_{0.5}$	1 bar, 298 K	-	44.58	10.75	42.5	4.14	21	[28]
$Co_2(dobdc)$	1 bar, 298 K	1438[a]	164.19	120.51	48	1.36	46	[13]
$Mg_2(dobdc)$	1 bar, 318 K	1797[a]	188.16	158.37	46	1.19	7.5	[13]
$Fe_2(dobdc)$	1 bar, 318 K	1536[a]	158.76	135.97	51	1.16	14.7	[29]
Cu-BTC	1 bar, 318 K	338	141.12	112	50	1.26	6.1	[30]
Zu-36-Ni	1 bar, 298 K	246	44.8	11.2	42	4	1.8	[20]
GeFSIX-2-Cu-i	1 bar, 298 K	467	60.26	40.31	36.5	1.49	4	[31]
SiFSIX-2-Cu-i	1 bar, 298 K	503	59.36	37.41	37	1.59	4.5	[31]

Zeolite 13X	1 bar, 318 K	-	47.01	35.85	-	1.31	8.2	[32]
CPL-1	1 bar, 273 K	330	39.87	6.72	-	5.93	2.08	[33]
HOF-16	1 bar, 298 K	279	58.69	33.15	32.9	1.77	5.5	[34]
ITQ-12	1 bar, 303 K	-	29.12	16.8	-	1.73	15	[35]
Fe_2(m-dobdc)	1 bar, 298 K	-	163.74	132.61	73	1.23	60	[19]

Supplementary References

1. Wang J, Zhang M, Xu X, Feng J, Wang Y, Zhang M, Han W, Chen Y, Tian G. Synthesis and characterization of [Cu(N-MeIm)$_4$(BF$_4$)$_2$] in ionic liquid. *Chem. Res. Chin. Univ.* **34**, 8-12 (2018).
2. Cho H-G, Andrews L. Infrared Spectra of CH_3–MF and Several Fragments Prepared by Methyl Fluoride Reactions with Laser-Ablated Cu, Ag, and Au Atoms. *Inorg. Chem.* **50**, 10319-10327 (2011).
3. Trofimov BA, Sinegovskaya LM, Gusarova NK. Vibrations of the S–S bond in elemental sulfur and organic polysulfides: a structural guide. *J. Sulfur Chem.* **30**, 518-554 (2009).
4. Ibrahim I, Yunus S, Hashim M. Relative performance of isopropylamine, pyrrole and pyridine as corrosion inhibitors for carbon steels in saline water at mildly elevated temperatures. *Int. J. Sci. Eng. Res* **4**, 1-12 (2013).
5. Baik S, Zhang H, Kim YK, Harbottle D, Lee JW. Enhanced adsorption capacity and selectivity towards strontium ions in aqueous systems by sulfonation of CO_2 derived porous carbon. *RSC Adv.* **7**, 54546-54553 (2017).
6. Campos-Vallette M, Clavijo R, Mendizabal F, Zamudio W, Baraona R, Diaz G. Infrared spectrum of the bis-(1, 10-phenanthroline) Cu (I) and Cu (II) perchlorate complexes. *Vib. Spectrosc.* **12**, 37-44 (1996).
7. Bao Z, Wang J, Zhang Z, Xing H, Yang Q, Yang Y, Wu H, Krishna R, Zhou W, Chen B, Ren Q. Molecular Sieving of Ethane from Ethylene through the Molecular Cross-Section Size Differentiation in Gallate-based Metal–Organic Frameworks. *Angew. Chem. Int. Ed.* **57**, 16020-16025 (2018).
8. Yang S, Ramirez-Cuesta AJ, Newby R, Garcia-Sakai V, Manuel P, Callear SK, Campbell SI, Tang CC, Schröder M. Supramolecular binding and separation of hydrocarbons within a functionalized porous metal–organic framework. *Nat. Chem.* **7**, 121-129 (2015).
9. Lin R-B, Li L, Zhou H-L, Wu H, He C, Li S, Krishna R, Li J, Zhou W, Chen B. Molecular sieving of ethylene from ethane using a rigid metal–organic framework. *Nat. Mater.* **17**, 1128-1133 (2018).
10. Yang Y, Li L, Lin R-B, Ye Y, Yao Z, Yang L, Xiang F, Chen S, Zhang Z, Xiang S, Chen B. Ethylene/ethane separation in a stable hydrogen-bonded organic framework through a gating mechanism. *Nat. Chem.* **13**, 933-939 (2021).
11. Zhang L, Li L, Hu E, Yang L, Shao K, Yao L, Jiang K, Cui Y, Yang Y, Li B, Chen B, Qian G. Boosting Ethylene/Ethane Separation within Copper(I)-Chelated Metal–Organic Frameworks through Tailor-Made Aperture and Specific π-Complexation. *Adv. Sci.* **7**, 1901918 (2020).
12. Mofarahi M, Salehi SM. Pure and binary adsorption isotherms of ethylene and ethane on zeolite 5A. *Adsorption* **19**, 101-110 (2013).
13. He Y, Krishna R, Chen B. Metal–organic frameworks with potential for energy-efficient adsorptive separation of light hydrocarbons. *Energy Environ. Sci.* **5**, 9107-9120 (2012).
14. Ding Q, Zhang Z, Yu C, Zhang P, Wang J, Cui X, He C-H, Deng S, Xing H. Exploiting equilibrium-kinetic synergetic effect for separation of ethylene and ethane in a microporous metal-organic framework. *Sci. Adv.* **6**, eaaz4322 (2020).
15. Wang Y, Yuan S, Hu Z, Kundu T, Zhang J, Peh SB, Cheng Y, Dong J, Yuan D, Zhou H-C, Zhao D. Pore Size Reduction in Zirconium Metal–Organic Frameworks for Ethylene/Ethane Separation. *ACS Sustainable Chem. Eng.* **7**, 7118-7126 (2019).
16. Li B, Zhang Y, Krishna R, Yao K, Han Y, Wu Z, Ma D, Shi Z, Pham T, Space B, Liu J, Thallapally PK, Liu J, Chrzanowski M, Ma S. Introduction of π-Complexation into Porous Aromatic Framework for Highly Selective Adsorption of Ethylene over Ethane. *J. Am. Chem. Soc.* **136**, 8654-8660 (2014).
17. Anson A, Wang Y, Lin CCH, Kuznicki TM, Kuznicki SM. Adsorption of ethane and ethylene on modified ETS-10. *Chem. Eng. Sci.* **63**, 4171-4175 (2008).
18. Chuah CY, Samarasinghe S, Li W, Goh K, Bae TH. Leveraging Nanocrystal HKUST-1 in Mixed-Matrix

Membranes for Ethylene/Ethane Separation. *Membranes (Basel)* **10**, (2020).

19. Bachman JE, Kapelewski MT, Reed DA, Gonzalez MI, Long JR. M_2(m-dobdc) (M = Mn, Fe, Co, Ni) Metal–Organic Frameworks as Highly Selective, High-Capacity Adsorbents for Olefin/Paraffin Separations. *J. Am. Chem. Soc.* **139**, 15363-15370 (2017).
20. Zhang Z, Ding Q, Cui X, Jiang X-M, Xing H. Fine-Tuning and Selective-Binding within an Anion-Functionalized Ultramicroporous Metal–Organic Framework for Efficient Olefin/Paraffin Separation. *ACS Appl Mater Inter* **12**, 40229-40235 (2020).
21. Bereciartua PJ, Cantín Á, Corma A, Jordá JL, Palomino M, Rey F, Valencia S, Corcoran EW, Kortunov P, Ravikovitch PI, Burton A, Yoon C, Wang Y, Paur C, Guzman J, Bishop AR, Casty GL. Control of zeolite framework flexibility and pore topology for separation of ethane and ethylene. *Science* **358**, 1068-1071 (2017).
22. Liang B, Zhang X, Xie Y, Lin R-B, Krishna R, Cui H, Li Z, Shi Y, Wu H, Zhou W, Chen B. An Ultramicroporous Metal–Organic Framework for High Sieving Separation of Propylene from Propane. *J. Am. Chem. Soc.* **142**, 17795-17801 (2020).
23. Cadiau A, Adil K, Bhatt PM, Belmabkhout Y, Eddaoudi M. A metal-organic framework–based splitter for separating propylene from propane. *Science* **353**, 137-140 (2016).
24. Wang H, Dong X, Colombo V, Wang Q, Liu Y, Liu W, Wang X-L, Huang X-Y, Proserpio DM, Sironi A, Han Y, Li J. Tailor-Made Microporous Metal–Organic Frameworks for the Full Separation of Propane from Propylene Through Selective Size Exclusion. *Adv. Mater.* **30**, 1805088 (2018).
25. Yu L, Han X, Wang H, Ullah S, Xia Q, Li W, Li J, da Silva I, Manuel P, Rudić S, Cheng Y, Yang S, Thonhauser T, Li J. Pore Distortion in a Metal–Organic Framework for Regulated Separation of Propane and Propylene. *J. Am. Chem. Soc.* **143**, 19300-19305 (2021).
26. Zeng H, Xie M, Wang T, Wei R-J, Xie X-J, Zhao Y, Lu W, Li D. Orthogonal-array dynamic molecular sieving of propylene/propane mixtures. *Nature* **595**, 542-548 (2021).
27. Liu T, Cui H, Zhang X, Zhang Z-Y, Lin R-B, Liang B, Zhang J, Li D, Chen B. Doubly Interpenetrated Metal–Organic Framework of pcu Topology for Selective Separation of Propylene from Propane. *ACS Appl Mater Inter* **12**, 48712-48717 (2020).
28. Wu H, Yuan Y, Chen Y, Xu F, Lv D, Wu Y, Li Z, Xia Q. Efficient adsorptive separation of propene over propane through a pillar-layer cobalt-based metal–organic framework. *AlChE J.* **66**, e16858 (2020).
29. Bloch ED, Queen WL, Krishna R, Zadrozny JM, Brown CM, Long JR. Hydrocarbon Separations in a Metal-Organic Framework with Open Iron(II) Coordination Sites. *Science* **335**, 1606-1610 (2012).
30. Ferreira AFP, Santos JC, Plaza MG, Lamia N, Loureiro JM, Rodrigues AE. Suitability of Cu-BTC extrudates for propane–propylene separation by adsorption processes. *Chem. Eng. J.* **167**, 1-12 (2011).
31. Wang X, Zhang P, Zhang Z, Yang L, Ding Q, Cui X, Wang J, Xing H. Efficient Separation of Propene and Propane Using Anion-Pillared Metal–Organic Frameworks. *Ind. Eng. Chem. Res.* **59**, 3531-3537 (2020).
32. Campo MC, Baptista MC, Ribeiro AM, Ferreira A, Santos JC, Lutz C, Loureiro JM, Rodrigues AE. Gas phase SMB for propane/propylene separation using enhanced 13X zeolite beads. *Adsorption* **20**, 61-75 (2014).
33. Chen Y, Qiao Z, Lv D, Duan C, Sun X, Wu H, Shi R, Xia Q, Li Z. Efficient adsorptive separation of C_3H_6 over C_3H_8 on flexible and thermoresponsive CPL-1. *Chem. Eng. J.* **328**, 360-367 (2017).
34. Gao J, Cai Y, Qian X, Liu P, Wu H, Zhou W, Liu D-X, Li L, Lin R-B, Chen B. A Microporous Hydrogen-Bonded Organic Framework for the Efficient Capture and Purification of Propylene. *Angew. Chem. Int. Ed.* **60**, 20400-20406 (2021).
35. Gutiérrez-Sevillano JJ, Dubbeldam D, Rey F, Valencia S, Palomino M, Martín-Calvo A, Calero S. Analysis of the ITQ-12 Zeolite Performance in Propane−Propylene Separations Using a Combination of Experiments and Molecular Simulations. *The Journal of Physical Chemistry C* **114**, 14907-14914 (2010).
